# Genetic feature engineering enables characterisation of shared risk factors in immune-mediated diseases

**DOI:** 10.1186/s13073-020-00797-4

**Published:** 2020-11-25

**Authors:** Oliver S. Burren, Guillermo Reales, Limy Wong, John Bowes, James C. Lee, Anne Barton, Paul A. Lyons, Kenneth G. C. Smith, Wendy Thomson, Paul D. W. Kirk, Chris Wallace

**Affiliations:** 1 Cambridge Institute of Therapeutic Immunology & Infectious Disease (CITIID), Jeffrey Cheah Biomedical Centre, Cambridge Biomedical Campus, University of Cambridge, Puddicombe Way, Cambridge, CB2 0AW UK; 2 Department of Medicine, University of Cambridge School of Clinical Medicine, Cambridge Biomedical Campus, Cambridge, CB2 0QQ UK; 3 National Institute of Health Research Manchester Biomedical Research Centre, Manchester Academic Health Science Centre, Manchester University NHS Foundation Trust, Manchester, UK; 4 Centre for Genetics and Genomics Versus Arthritis, Centre for Musculoskeletal Research, The University of Manchester, Manchester, UK; 5 MRC Biostatistics Unit, University of Cambridge, Forvie Site, Cambridge Biomedical Campus, Cambridge, CB2 0SR UK; 6 Cancer Research UK Cambridge Centre, Ovarian Cancer Programme, University of Cambridge Li Ka Shing Centre, Robinson Way, Cambridge, CB2 0RE UK

## Abstract

**Background:**

Genome-wide association studies (GWAS) have identified pervasive sharing of genetic architectures across multiple immune-mediated diseases (IMD). By learning the genetic basis of IMD risk from common diseases, this sharing can be exploited to enable analysis of less frequent IMD where, due to limited sample size, traditional GWAS techniques are challenging.

**Methods:**

Exploiting ideas from Bayesian genetic fine-mapping, we developed a disease-focused shrinkage approach to allow us to distill genetic risk components from GWAS summary statistics for a set of related diseases. We applied this technique to 13 larger GWAS of common IMD, deriving a reduced dimension “basis” that summarised the multidimensional components of genetic risk. We used independent datasets including the UK Biobank to assess the performance of the basis and characterise individual axes. Finally, we projected summary GWAS data for smaller IMD studies, with less than 1000 cases, to assess whether the approach was able to provide additional insights into genetic architecture of less common IMD or IMD subtypes, where cohort collection is challenging.

**Results:**

We identified 13 IMD genetic risk components. The projection of independent UK Biobank data demonstrated the IMD specificity and accuracy of the basis even for traits with very limited case-size (e.g. vitiligo, 150 cases). Projection of additional IMD-relevant studies allowed us to add biological interpretation to specific components, e.g. related to raised eosinophil counts in blood and serum concentration of the chemokine CXCL10 (IP-10). On application to 22 rare IMD and IMD subtypes, we were able to not only highlight subtype-discriminating axes (e.g. for juvenile idiopathic arthritis) but also suggest eight novel genetic associations.

**Conclusions:**

Requiring only summary-level data, our unsupervised approach allows the genetic architectures across any range of clinically related traits to be characterised in fewer dimensions. This facilitates the analysis of studies with modest sample size by matching shared axes of both genetic and biological risk across a wider disease domain, and provides an evidence base for possible therapeutic repurposing opportunities.

## Background

The collected summary data of genome-wide association studies (GWAS) represent, in a compressed form, assays of thousands of phenotypes across millions of common genetic variants. Analysed individually, GWAS have elucidated the polygenic component of common human diseases [1], and comparative studies of summary GWAS results have highlighted a shared genetic aetiology across different diseases [2]. Evidence for such sharing can highlight opportunities for therapeutic repurposing [3]. However, comprehensive overviews of sharing between multiple diseases are made difficult by the dimension of these statistics (100,000s of SNPs), the complex patterns that exist, and the limitation that while all dimensions carry information about technical differences between studies (DNA storage, processing, and population sampling), only a minority carry information about disease risk. Therefore, integrative analyses have typically been approached from one of two angles: a variant-by-variant analysis across multiple diseases focusing on individual variants in turn [4, 5], or pairwise analysis of diseases across multiple variants at a regional or genome-wide level [6, 7]. Both approaches have limitations. Different variants reflect different patterns of sharing across diseases, making generalisations about inter-disease relationships difficult, while disease-pairwise approaches make comparison of more than two diseases challenging. Thus, a need exists for a framework to study shared genetic architectures across multiple variants and between multiple diseases simultaneously.

The GWAS approach explicitly accounts for the number of tests (SNPs) by requiring successively larger samples (tens of thousands). Large samples present an insurmountable barrier for rare diseases, where efforts have instead focused on searching for rare variants of high penetrance through whole exome [8] or whole genome [9, 10] sequencing. Despite this, moderate-sized GWAS-style studies of rare diseases have found both polygenic association with common variants [10, 11] and evidence for differential genetic associations between clinical subtypes of these rare diseases [12]. Thus, a need exists to democratise GWAS to less common diseases, which may be possible by considering them in the context of more common, clinically related diseases.

We propose summarising the multifactorial genetic risks of related diseases in an informed dimension-reduction approach. Matrix decomposition, for example via principal component analysis (PCA), expresses a matrix as the product of two smaller matrices and has been used extensively as a dimension-reduction tool in genetics to summarise population structure and address its confounding effects in association studies [13]. It has also been used to explore structure in genetic association with multiple traits, either from different studies aggregating signals across nearby SNPs [14], or using a linkage disequilibrium (LD) independent subset of SNPs from a single cohort [15]. In either case, the reduced dimensional space was used to explore the same datasets as used to define it, with two implications. First, GWAS summary statistics are a composite of biological signal, technical noise, and sampling variation. Decomposition aims to find axes that maximise variance explained in the input datasets, and cannot distinguish between these three sources of variability. We therefore expect it to magnify technical and random differences as well as biological, a problem related to overfitting in high-dimensional datasets. Second, in this reduced dimension space, there is no treatment of uncertainty, so while we can measure the distance between diseases, we are unable to formally assess whether that distance significantly differs from 0.

Here, we propose augmenting PCA of GWAS summary statistics by a Bayesian shrinkage approach that mitigates overfitting. Our central aim is to define a reduced dimension space, with components that describe different patterns of genetic susceptibility corresponding to underlying biological risk factors. In a transfer learning paradigm, we can project independent datasets into this space, allowing us to study the distinct and shared genetic contributions to related diseases, and use standard statistical techniques to test for genetic association of rare diseases or genetic differences between disease subtypes. We use immune-mediated diseases (IMD) as an example of a set of traits with established aetiological overlap [2] to highlight the potential uses of this method.

## Methods

### Method for constructing a common genetic basis for related diseases

We aimed to decompose common components underlying susceptibility to a set of related diseases using PCA. There are three particular challenges with performing PCA on GWAS summary statistics. First, the SNP effect estimates (e.g. log odds ratios, denoted $\hat{\beta}$) must be on the same scale; second, we must deal with variable correlation between input dimensions (SNPs) due to LD; and third, while all SNPs are expected to show small deviations between studies due to random noise, different genotyping platforms, and data processing decisions, only a minority of SNPs will be truly related to the diseases of interest.

The uncertainty attached to $\hat{\beta}$ depends on both study sample size and SNP minor allele frequency (MAF). We adjusted for the variance in $\hat{\beta}$ due to MAF, *σ*^2^_*MAF*_, as this varies between SNPs, but not variance due to sample size, as this would overly shrink smaller studies relative to larger. To ensure the disease relevance of the basis, we wanted to preferentially use information from truly associated SNPs, while avoiding double counting evidence from SNPs in LD. We therefore dealt with the latter two challenges simultaneously, using a Bayesian fine-mapping technique which calculates the “posterior probability” that each SNP is causal for each trait, under the assumption that at most one causal variant exists in each recombination hotspot-defined block of SNPs [16, 17]. Note that the method also assumes the causal variant is in the dataset, an assumption likely to be violated without dense GWAS data. We thus use the method not to interpret the output as genuine probabilities, but for its side effect of generating a shrinkage weight that naturally adjusts for LD. At each SNP, we computed a weighted average of the posterior probabilities across input studies to create an overall weight for that SNP, *w*. *w* will be close to zero when there is no association in a region, limiting the influence of technical noise between studies, and will otherwise act to weight associated SNPs according to the extent of LD in a region. The final input for basis creation is a matrix of $\hat{\gamma}=w\hat{\beta}/{\sigma}_{MAF}$.

**Fig. 1:**
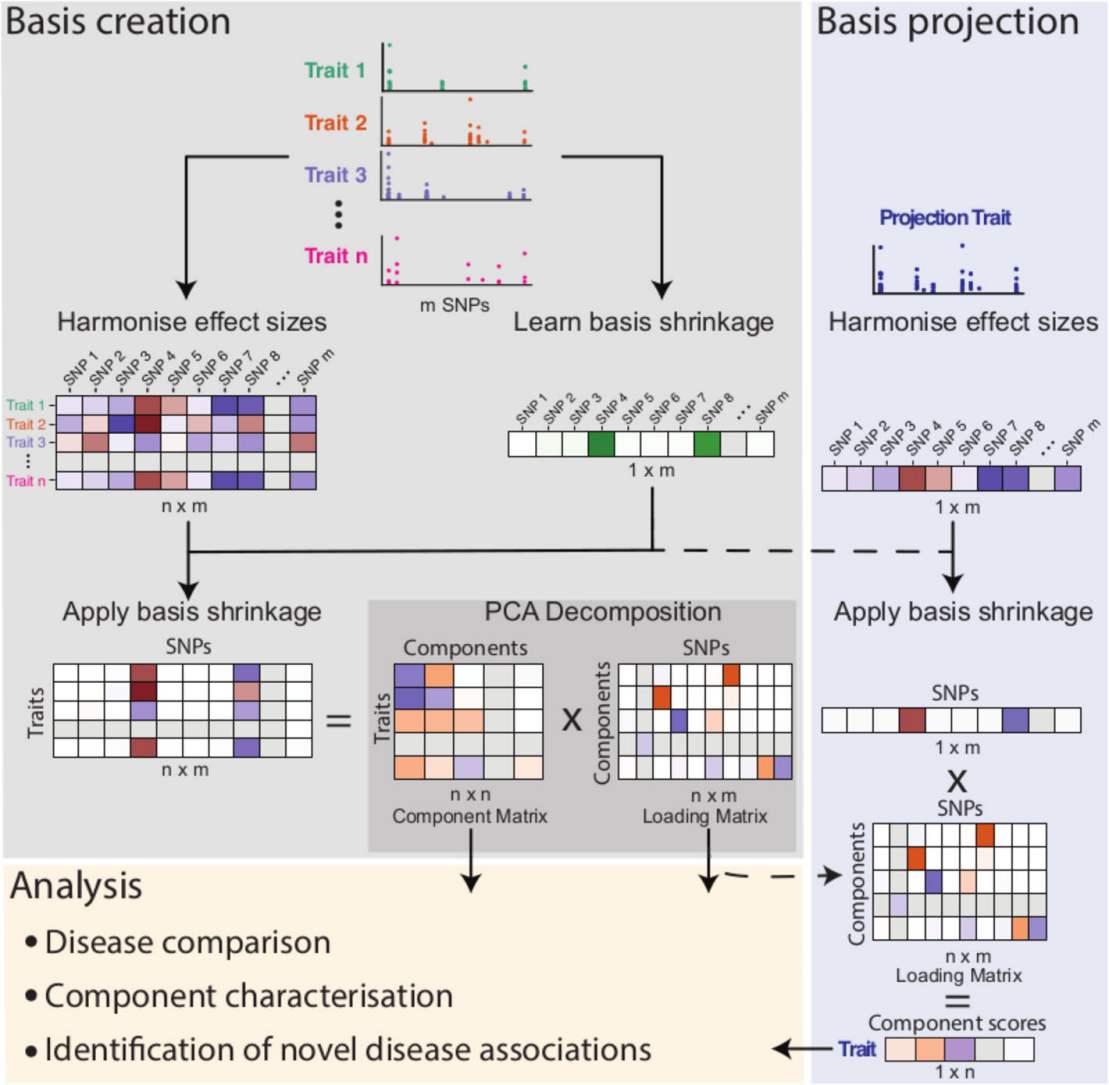
Schematic of basis creation and projection. Basis creation: GWAS summary statistics for related traits are combined to create a matrix, *M* (*n* × *m*), of harmonised effect sizes ($\hat{\beta}$) and a learnt vector of shrinkage values for each SNP. After multiplying each row of *M* by the shrinkage vector, PCA is used to decompose *M* into component and loading matrices. Basis projection: for an independent set of studies, trait effects are harmonised with respect to the basis, shrinkage applied, and the resultant vector is multiplied by the basis loading matrix to obtain component scores. These component scores can be used for testing hypotheses of the form that a weighted average of effect sizes in the test GWAS is non-zero, because the weights (basis loading matrix) are learnt from an independent set of large GWAS

A mathematically detailed summary is given in Additional File 1, and a summary of the method is shown in Fig. 1.

### Construction of IMD basis

We identified 13 IMD GWAS with > 6000 samples of European ancestry for which full summary statistics were publicly available (Additional File 2: Table S1). Studies were chosen to balance the competing aims of maximising the number of studies, the number of SNPs common to all studies, and the number of samples in each study (to minimise noise in $\hat{\beta}$). We selected SNPs present in all 13 studies, with MAF > 1% in the 1000 Genomes Phase 3 EUR data. We excluded all variants within the major histocompatibility complex (MHC, GRCh37 Chr6:20-40Mb) due to its long and complex LD structure, and because SNPs in the MHC have a profound involvement in IMD susceptibility, and thus the potential to dominate the basis. We also excluded SNPs for which the unambiguous assignment of the effect allele was impossible (e.g. palindromic SNPs). We harmonised all effect estimates to be with respect to the alternative allele relative to the reference allele as defined by the 1000 Genomes reference genotype panel. After filtering, harmonised effect estimates were available for 265,887 SNPs across all 13 selected “basis” traits (Additional File 3: Fig. S1), and additional analyses of a subset of six datasets with dense genotyping showed that these 265,887 SNPs adequately tagged the information available in the full SNP data (Additional File 1). In order to provide a baseline for subsequent analyses, we created an additional synthetic control trait, for which effect sizes across all traits were set to zero. This can be thought of as the limit of a simulated null GWAS as the number of cases and controls tends to infinity (Additional File 1). We used these to construct two matrices *M* and *M*^′^ where elements reflect raw ($\hat{\beta}$) and shrunk effect sizes ($\hat{\gamma}=w\hat{\beta}/{\sigma}_{MAF}$), respectively, such that rows and columns reflect traits (*n* = 14) and SNPs (*p* = 265,887). After mean centring columns, we used the R command *prcomp* to carry out PCA of both *M* and *M*^′^ to generate naive and “shrunk” IMD bases. It is likely that the trailing components of any PCA represent noise, so to assess the maximal subset of informative components, we examined the mean squared reconstruction error and found that the fewest components needed to minimise this error were *m = n* − 1 = 13 (Additional File 3: Fig. S2). We therefore discarded the final 14th component. As in conventional PCA, this basis consists of orthogonal principal components (PCs), constructed as linear functions of input $\hat{\beta}$, which together provide a lower dimensional representation of genetic associations with IMD.

### Driver SNPs

We noted that the majority of entries in the *p* × *m* PCA rotation matrix, *Q*, were close to 0, and chose to hard threshold these to 0 for computational efficiency and to identify which *driver SNPs* were relevant to each component. To do this, using *Q*_*k*_ to represent the *k*th column of *Q*, we define *Q*_*k*_ (α) = *Q*_*k*_ x *I* (|*Q*_*k*_|>α) where *I* () is an indicator function and “x” represents element-wise multiplication. We quantify the distance between projection with *Q*_*k*_ and *Q*_*k*_*(*α) by:

$$ {D}_k\ \left(\upalpha \right)=1-\mathrm{cor}\ \left({M}^c\ {Q}_k,{M}^c\ {Q}_k\ \left(\upalpha \right)\right). $$

where *M*^*C*^ is the centred matrix of shrunk effect sizes *M*^′^, defined above. We chose the threshold for each component, α_*k*_, as the largest value α such that *D*_*k*_ (α) < 0.001. Finally, we defined the sparse basis rotation matrix as the matrix constructed from the column vectors *Q*_*k*_, *k* = 1,...,*m*. This identified both driver SNPs which define the support for each component, and enabled computationally efficient examination of many traits in the reduced dimension space defined.

### Projection of independent datasets

We constructed a compendium of publicly available GWAS summary statistics across a wide range of traits including UK Biobank (UKBB) self-reported traits (http://www.nealelab.is/uk-biobank, http://geneatlas.roslin.ed.ac.uk/—Additional File 2: Tables S2-S3), IMD-relevant GWAS (Additional File 2: Table S4), and GWAS of quantitative measures from blood count data [18], immune cell counts [19], and cytokine levels [20] (Additional File 2: Tables S5-S7). Disease GWAS data were obtained from the URL given or via request to study authors, with the exception of anti-neutrophil cytoplasmic antibody (ANCA)-associated vasculitis (AAV), juvenile idiopathic arthritis (JIA), and psoriatic arthritis (PsA) which are described in Additional File 4 and data given in Additional File 5, Table S9.

Prior to projection, effect alleles were aligned to the 1000 Genomes reference genotype panel. For traits sensitive to missing data (studies of neuromyelitis optica (NMO) [10], and 8 by Aterido [21] see Additional File 1), we imputed missing variants using ssimp [22] (v 0.5.6 --ref 1KG/EUR --impute.maf 0.01); otherwise, we set effect estimates to zero. Data were then shrunk as for the basis traits (multiplying by *w*/*σ*_*MAF*_), and projected into basis space by multiplying by the sparse basis rotation matrix *Q*. We report projected results as $\hat{\delta}$, the difference between the projected $\hat{\beta}$ and a projected synthetic control with all entries 0, which allows us to make statistical inference about whether its estimand, *δ*, differs from control. We calculated variance of $\hat{\delta}$ as described in Additional File 1.

GWAS test multiple null hypotheses of the form *β* = 0 to identify disease-associated SNPs. This approach has been extended to test genetic correlation through cross-trait polygenic score tests. A SNP set and weights are learnt to optimise genetic prediction of a trait of interest, and the weighted sums of *β* are constructed in a second dataset, and tested for association with a second trait of interest [23]. We consider each component in the basis to be a polygenic score for an uncharacterised factor contributing to one or more basis input traits. We looked for an association of the projected traits to any component by testing the null hypothesis that the vector *δ* = 0 across all 13 components using a chi-square test (Additional File 1: eq. 2). This null hypothesis is related to the global GWAS null hypothesis of no association, but is restricted to the small number of components identified in the basis, which are formed as a weighted linear function of a subset of variants. Failure to reject this null could reflect either a lack of power (as with all GWAS) or a lack of genetic association with the common components shared by the basis diseases. We called significant associations according to FDR < 0.01, calculated using the Benjamini-Hochberg approach, run independently within the broad categories: primary analysis (UKBB self-reported disease and cancer, plus IMD-relevant GWAS), blood cell counts, cytokines, and immune cell counts. This was our primary measure of significance. We took the same strategy to independently calculate FDR for each component individually for additional annotation, and traits were considered “component-significant” if they were significant (component FDR < 0.01) on that component *and* overall.

Classification of diseases according to autoantibody status was performed by a specialist clinician using available medical literature. This assignment was blinded to the PC1 results.

### Clustering

We used the *hclust ()* function in R to cluster diseases in the basis using agglomerative hierarchical clustering according to Ward’s criterion (method = “Ward.D2”) on the Euclidean distance between projected locations of each disease in the basis.

### Consistency

We would like to interpret significant results as representing a composite of many small effects working in consistent directions. However, false positives could also occur if a single SNP with a large weight in the basis is in LD with a SNP with a large effect on the projected trait due to chance. To guard against this, we used weighted Spearman rank correlation which is robust to such outlier observations to test the “consistency” of each projection on a subset of driver SNPs in low LD (*r*^2^ < 0.01), with weights w/σ_MAF_ and significance determined by permuting the projected values. All projected values are given in Additional File 6: Table S10.

### Candidate significant driver SNPs

For each of 10 diseases or subtypes with < 2000 cases and significant on at least one component (myasthenia gravis, late onset; eosinophilic granulomatosis with polyangiitis [EGPA], myeloperoxidase positive [MPO+], ANCA negative [ANCA−], and combined; JIA, extended oligoarticular [EO], persistent oligoarticular [PO], and polyarticular rheumatoid factor positive/negative [RF+ and RF−, respectively]), we selected all driver SNPs on any significant component and calculated the FDR within this set of SNPs as a subset-selected FDR [24].We ordered SNPs by increasing values of ssFDR and deleted any SNPs in the list that were in LD (*r*^2^ > 0.1) with a higher placed SNP, leaving a set of unlinked SNPs associated with each trait shown in Table 1. These were annotated through literature searches.

**Table 1:** Disease-associated SNPs identified through subset-selected FDR (ssFDR) < 0.01 amongst driver SNPs belonging to disease-significant components. Genes listed are nearby genes previously mentioned in the literature for the listed disease or basis diseases associated to this SNP, and are intended to indicate location; no evidence for gene causality has been assessed here. Where no basis diseases are associated with the SNP at genome-wide significant threshold (GWsig, *p* < 5 × 10^−8^), the strongest association and its *p* value are shown

Disease	SNP	Chrm	Position	*p* value	FDR	Genes	Basis diseases	Notes
*Genome-wide significant (4)*								
JIA RF−	rs2476601	1	114,377,568	2.36E−13	7.68E−11	*PTPN22*	CD, RA, SLE, T1D, VIT	
EGPA combined	rs13405741	2	111,913,056	2.89E−09	1.07E−06	*BCL2L11*	PSC	
EGPA ANCA−	rs11745587	5	131,796,922	3.59E−08	1.33E−05	*IRF1/IL5*	Asthma, CD	
JIA RF−	rs11065987	12	112,072,424	1.87E−08	2.81E−06	*SH2B3*	PBC, T1D, VIT	
*Genome-wide significant in another subtype or study (7)*								
JIA PO	rs2476601	1	114,377,568	7.59E−06	3.65E−03	*PTPN22*	CD, RA, SLE, T1D, VIT	RF− subtype of JIA
Myasthenia gravis combined	rs2476601	1	114,377,568	6.62E−05	2.61E−03	*PTPN22*	CD, RA, SLE, T1D, VIT	GWsig in myasthenia gravis
EGPA ANCA−	rs13405741	2	111,913,056	1.33E−06	2.46E−04	*BCL2L11*	PSC	GWsig in EGPA combined
JIA EO	rs7574865	2	191,964,633	7.77E−07	1.24E−04	*STAT4*	PBC, RA, SLE	GWsig in JIA combined
Myasthenia gravis combined	rs231804	2	204,708,646	8.57E−07	1.69E−04	*CTLA4*	RA, T1D	*r*^2^ > 0.5 with non-driver SNP rs231770, *p* = 3.98E−08
Myasthenia gravis late onset	rs231804	2	204,708,646	1.18E−05	2.33E−03	*CTLA4*	RA, T1D	*r*^2^ > 0.5 with non-driver SNP rs231770, *p* = 3.98E−08
JIA RF−	rs1893217	18	12,809,340	1.69E−06	1.10E−04	*PTPN2*	CD, RA, T1D	GWsig in JIA combined
*Supported by other evidence in another study (5)*								
EGPA combined	rs11745587	5	131,796,922	3.44E−07	6.38E−05	*IRF1*, *IL5*	Asthma, CD	GWsig conditional on asthma GWAS
EGPA combined	rs6454802	6	90,814,199	8.73E−06	6.48E−04	*BACH2*	Asthma, T1D, VIT	GWsig conditional on eosinophil count GWAS
EGPA ANCA−	rs6454802	6	90,814,199	1.23E−05	1.52E−03	*BACH2*	Asthma, T1D, VIT	GWsig conditional on eosinophil count GWAS
EGPA combined	rs8179	7	92,236,164	6.05E−06	5.61E−04	*CDK6*	RA 4.3e−07	GWsig conditional on eosinophil count GWAS
EGPA ANCA−	rs8179	7	92,236,164	5.51E−05	3.34E−03	*CDK6*	RA 4.3e−07	GWsig conditional on eosinophil count GWAS
*Not previously reported (8)*								
JIA RF−	rs9594746	13	42,989,660	1.06E−05	4.91E−04	*TNFSF11*	PBC 4.7e−07	*r*^2^ = 0.9 with rs34132030 (*p* = 2 × 10^−7^ in larger dataset [25])
EGPA combined	rs12405671	1	117,263,868	2.99E−06	3.70E−04	*CD2*, *CD28*	RA 1e−07	
EGPA ANCA−	rs12405671	1	117,263,868	4.06E−05	3.04E−03	*CD2*, *CD28*	RA 1e−07	
EGPA combined	rs1457115	5	110,567,598	3.21E−05	1.98E−03	*TSLP*, *WDR36*, *CAMK4*	Asthma	NB unlinked to nearby and previously reported EGPA-associated rs1837253 (*r*^2^ = 0.01)
EGPA ANCA−	rs1457115	5	110,567,598	2.16E−04	8.01E−03	*TSLP*, *WDR36*, *CAMK4*	Asthma	NB unlinked to nearby and previously reported EGPA-associated rs1837253 (*r*^2^ = 0.01)
Myasthenia gravis combined	rs2188962	5	131,770,805	3.78E−05	2.61E−03	*IRF1*, *IL5*	Asthma, CD	
Myasthenia gravis late onset	rs2188962	5	131,770,805	6.01E−05	5.95E−03	*IRF1*, *IL5*	Asthma, CD	
EGPA combined	rs10876864	12	56,401,085	1.19E−04	4.42E−03	*SUOX*, *IKZF4*	T1D, VIT	

## Results

### A genetic basis for immune-mediated diseases

**Fig. 2:**
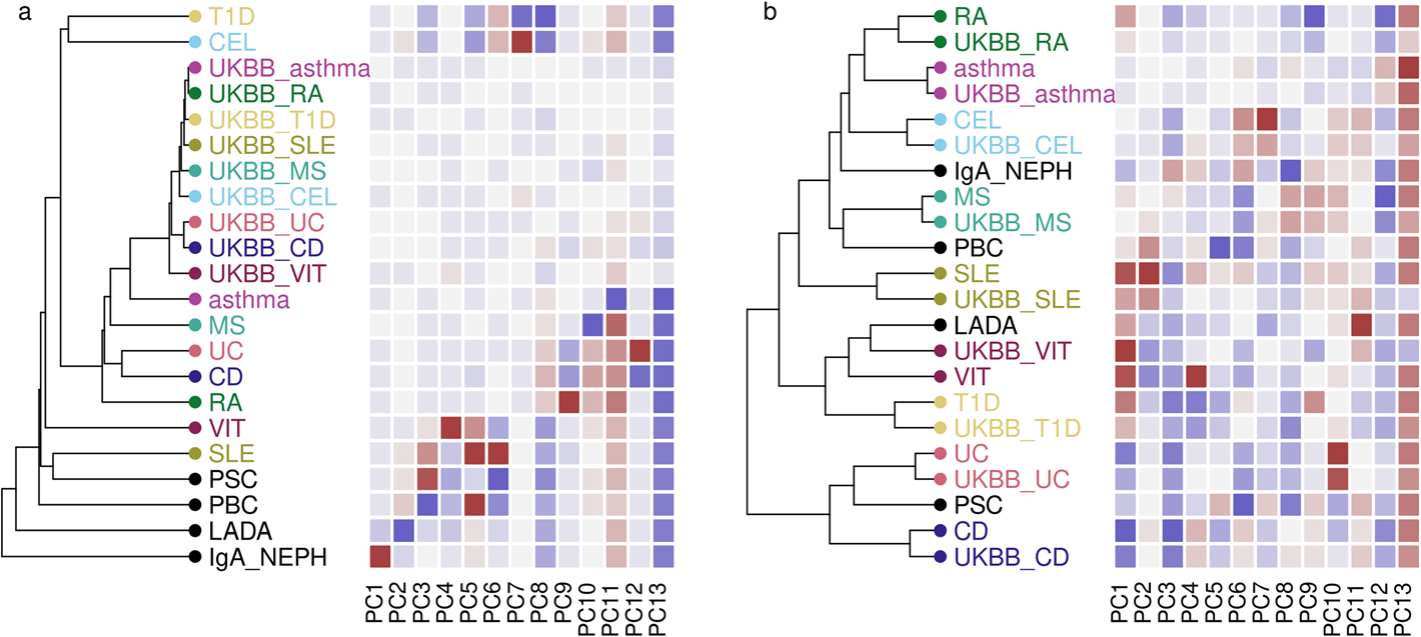
Hierarchical clustering of basis diseases and their UKBB counterparts in basis space. **a** Unweighted basis constructed using $\hat{\beta}$. **b** Basis constructed using continuous shrinkage applied to $\hat{\beta}$. Heatmaps indicate projected $\hat{\delta}$ for each disease on each component PC1–PC13, with grey indicating 0 (no difference from control), and darker shades of green or magenta showing departure from controls in one direction or the other. GWAS datasets: T1D, type 1 diabetes; CEL, celiac disease; asthma; MS, multiple sclerosis; UC, ulcerative colitis; CD, Crohn’s disease; RA, rheumatoid arthritis; VIT, vitiligo; SLE, systemic lupus erythematosus; PSC, primary sclerosing cholangitis; PBC, primary biliary cholangitis; LADA, latent autoimmune diabetes in adults; IgA_NEPH, IgA nephropathy. UKBB_ prefixed diseases correspond to self-reported disease status in UK Biobank

To illustrate the importance of our informed shrinkage procedure, we built four bases, with GWAS summary statistics for the 13 IMD shrunk differently in each case. We assessed their relative performance by projection of matching self-reported diseases (SRD) from UK BioBank (UKBB) [26] using summary statistics from a compendium provided by the Neale lab [http://www.nealelab.is/uk-biobank/], and used hierarchical clustering to examine whether expected patterns of similarities between diseases are captured in each reduced dimension space. The first was a naive approach without any shrinkage. Here, the UKBB SRD clustered with each other rather than their GWAS comparator, suggesting that the structure identified related to between-study differences other than disease (Fig. 2). In contrast, in the basis created with continuous shrinkage, all selected UKBB SRD clearly clustered with their GWAS comparators (Fig. 2), suggesting that the structure captured is disease-relevant, such that UKBB data from relatively infrequent diseases such as type 1 diabetes (T1D) (318 cases) and vitiligo (105 cases) are projected onto the same vectors as their larger comparator GWAS.

To illustrate the importance of using continuous shrinkage, we compared it to hard-thresholding, as used in the single-dataset decomposition approach, DeGAs [15], which replaced $\hat{\beta}$ by *Z* scores, and set *Z* = 0 when the associated *p* > 0.001. As *Z* scores are standardised $\hat{\beta}$, this has the effect of shrinking $\hat{\beta}$ towards 0 when uncertainty is high, such as when allele *or* disease frequencies are low, which means information from more common diseases will dominate. We generated hard-thresholded, LD-thinned bases using either *Z* scores or $\hat{\beta}$. For these, some of the structure identified was disease-related for the larger GWAS of more common traits (asthma, multiple sclerosis [MS], Crohn’s disease [CD], ulcerative colitis [UC]), but the smaller diseases were dominated by dataset-specific structure (Additional File 3: Fig. S3).

We projected data from three classes of study onto the basis with shrinkage. First, we used all self-reported disease and cancer traits from UKBB to characterise the basis components, to examine specificity to IMD, and to assess power as a function of sample size: case numbers for UKBB self-reported IMD range from 41,000 (asthma) to 105 (vitiligo). Second, we used IMD GWAS with smaller sample sizes than used in basis construction, including diseases studied in multiple ancestral backgrounds to explore robustness to ancestry differences. Third, we used the basis to analyse studies of IMD that are too rare or clinically heterogeneous to build large GWAS cohorts.

### Genetic analysis of multiple IMD in reduced dimensions

**Fig. 3:**
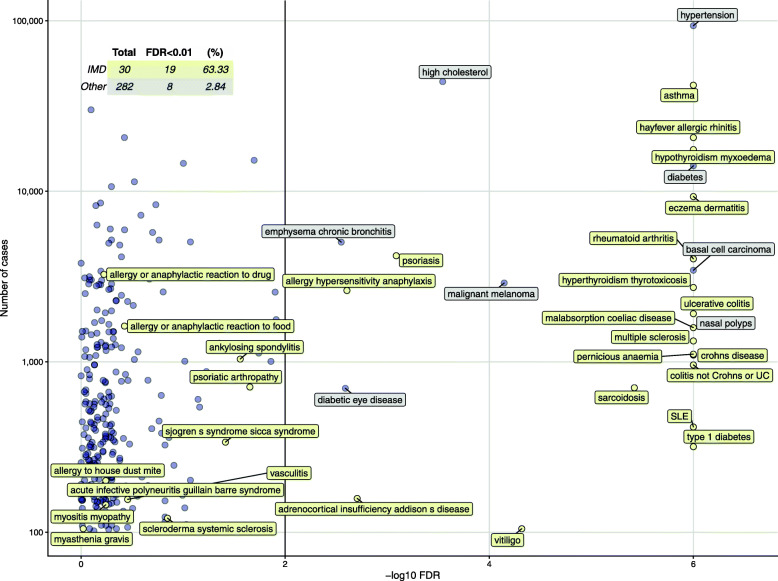
Of 312 UKBB self-reported traits projected onto the basis, 27 were significant at FDR < 1%, and IMD were enriched amongst this set, with 63% of IMD showing significance compared to < 3% of non-IMD traits. Each trait projected is shown according to FDR (−log10 scale, axis truncated at FDR = 10^−6^ for display) and number of cases. All IMD (yellow) and all significant non-IMD traits (grey) are labelled

Across all 312 projected UKBB traits (Additional File 2: Table S2), 27 had significantly non-zero $\hat{\boldsymbol{\delta}}$ (FDR < 1%). These were overwhelmingly immune-related traits (Fig. 3): no significance was observed for traits such as coronary artery disease, stroke, or obstructive sleep apnoea, confirming the immune-mediated specificity of our basis. Significant results were detected with as few as 105 cases for vitiligo, emphasising the potential of this approach to unlock the genetics of rare IMD GWAS.

Of 28 traits from target (non-UKBB) IMD GWAS, including JIA, NMO, vasculitis, and their clinical subtypes, 16 were significant (FDR < 1%, Additional File 2: Table S3, Additional File 3: Fig. S4-S16). We found, reassuringly, that increasing evidence for non-zero *δ* on any component correlated with increasing consistency on that component (see the “Methods” section) amongst disease traits (Additional File 3: Fig. S17), suggesting that significant results were produced by an average effect over many driver SNPs rather than random overlap of a small number of driver SNPs with trait-associated SNPs.

**Fig. 4:**
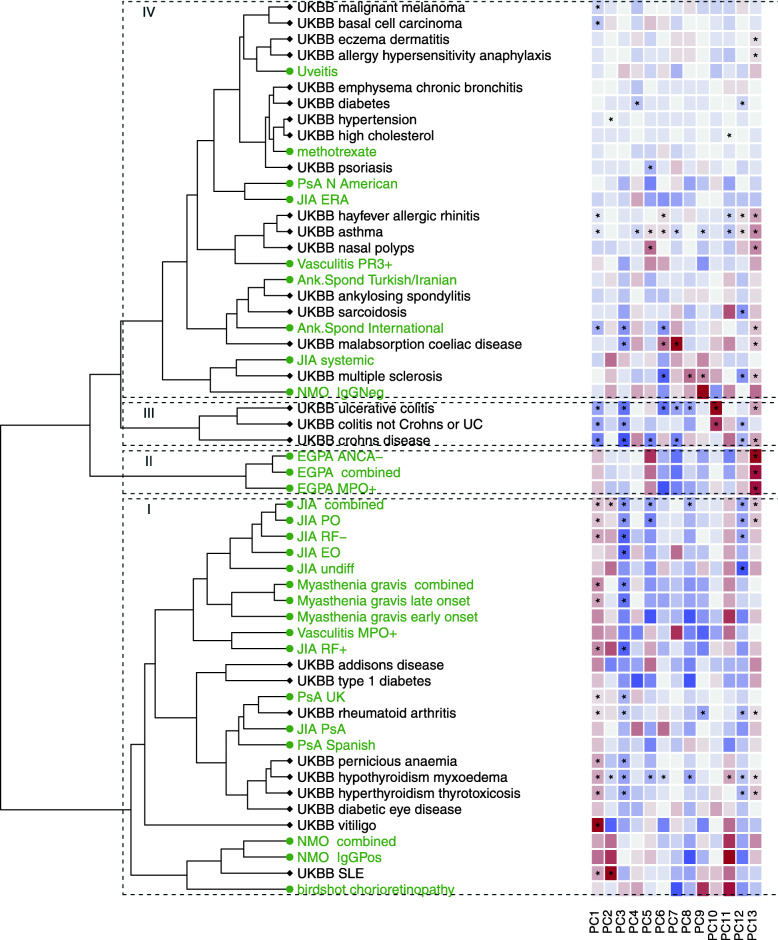
Hierarchical clustering of projected diseases significantly different from control (FDR < 1%) or of small sample size. Coloured labels are used to distinguish UKBB (grey) and other GWAS (green) datasets. Heatmaps indicate delta values for each disease on each component PC1–PC13, with grey indicating 0 (no difference from control), and darker shades of blue or magenta showing departure from controls in one direction or the other. An overlaid “*” indicates delta was significantly non-zero (FDR < 1%). Roman numerals indicate clusters described in the text. ANCA−, anti-neutrophil cytoplasmic antibody negative; Ank. Spond, ankylosing spondylitis; EGPA, eosinophilic granulomatosis with polyangiitis; EO, extended oligo; ERA, juvenile enthesitis-related arthritis; IgGPos, IgG positive; JIA, juvenile idiopathic arthritis; MPO+, myeloperoxidase positive; NMO, neuromyelitis optica; PO, persistent oligo; PR3+, proteinase 3 positive; PsA, psoriatic arthritis; RF+/−, polyarticular rheumatoid factor positive/negative; SLE, systemic lupus erythematosus; UC, ulcerative colitis

We clustered all 28 target traits and all 27 significant UKBB self-reported traits to generate a visual overview of IMD and associated traits (Fig. 4). Hierarchical clustering solutions are generally unstable and dependent on the composition of the items to be clustered, as well as the method used for clustering [27]. While clustering provides only a visual overview rather than a formal statistical analysis of trait similarity, it highlighted two small disease groups, inflammatory bowel disease (IBD) and EGPA, and two larger groups, one comprising autoimmune diseases and the other a heterogeneous cluster containing subgroups centred on MS, ankylosing spondylitis (AS), atopy, and traits with only weak or non-significant signals. Notably, three studies of AS all clustered together, despite only one having sufficient sample size for significant results and the three studies representing different ancestries (UK-European, International, and Turkish/Iranian).

While our basis was created from predominantly European GWAS, there is an imperative to increase ancestry diversity in GWAS [28]. We undertook a search for available IMD GWAS data with coverage of non-European ancestry and identified 6 studies of asthma, RA, UC, and CD in African and/or East Asian ancestry populations (Additional File 2: Table S8). Projecting these onto the basis, we find that all significant points have the same sign of delta for any given ancestry and PC combination (Additional File 3: Fig. S18). Thus, results are consistent across GWAS of the same traits in populations with different ancestry backgrounds. A broader examination comparing projections of all ~ 452,000 UKBB subjects to the European subset of 360,000 subjects found that while the mixed ancestry GWAS tended to result in slightly attenuated estimates of $\hat{\delta}$, the increased sample size also led to increased power compared to smaller European GWAS (Additional File 3: Fig. S19).

Most disease subtypes clustered together (Fig. 4). For example, myasthenia gravis, a chronic, autoimmune, neuromuscular disease characterised by muscle weakness, has been shown to have a bimodal incidence pattern by age, and some genetic associations have been identified only for the late-onset subtype [29]. However, both subtypes fell in very similar locations across all components and cluster together with several subtypes of JIA.

For two other diseases, however, subtypes clustered apart. NMO is a rare (prevalence 0.03–0.4:10,000) disease affecting the optic nerve and spinal cord for which HLA association is established [10] and which can be divided according to aquaporin 4 autoantibody seropositivity status (IgG+ or IgG−). The projections of seropositive and seronegative NMO showed non-significant differences on several components, leading to differential clustering. While seropositive NMO clustered with the classical autoimmune diseases, most closely with systemic lupus erythematosus [SLE] and Sjögren’s disease, IgG− NMO clustered away from the classic seropositive diseases, most closely with MS. This finding mirrors analysis which directly compared NMO subtypes to each of SLE and MS via polygenic scores [10], and strengthens the findings by specifically suggesting SLE and MS as the nearest neighbours of IgG+ and IgG− NMO, respectively, out of all IMD considered for clustering.

JIA is a heterogeneous paediatric disease, with an overall childhood prevalence in Europe of 20/10,000 [30], and with seven recognised subtypes [31]. While studies have begun to identify distinct genetics of the systemic subtype [32] and have shown subtype-specific differences in the MHC [33], systematic comparison between subtypes has been underpowered. Although, the systemic and enthesitis-related arthritis (ERA) subtypes did not significantly differ from controls (despite relatively moderate sample sizes of 219 and 267 cases, respectively), they clustered with MS and AS, respectively, and away from the other JIA subtypes, which clustered with the other autoimmune diseases.

### Association of driver SNPs to rare IMD or subtypes

Given that most of the IMD and subtypes with small GWAS have few established genetic associations, we sought to exploit the component-level associations above to detect new disease associations. Our basis has only 13 dimensions. If genetic susceptibility to rare IMD and IMD subtypes overlaps that of common IMD, we can increase power by focusing on these dimensions. Of 22 diseases or disease subtypes with < 1000 cases, 12 were significant (FDR < 1%), even with as few as 132 cases (NMO IgG+).

Although not a specific goal, the basis generated is naturally sparse (Additional File 3: Fig. S20), enabling us to identify 107–373 “driver SNPs” that are required to capture genetic associations on any individual component. We found a strong enrichment for small GWAS *p* values at driver SNPs on trait-significant components (Additional File 3: Fig. S21). Using a “subset-selected” FDR approach [24], we analysed driver SNPs for 22 significant trait-component pairs (12 unique traits) and identified 25 trait-SNP associations (subset-selected FDR < 1%, Table 1) after pruning SNPs in LD. Twelve of these were genome-wide significant (*p* < 5 × 10^−8^) either in this study (4 associations) or in other published data (8 associations), and a further five were significant in other published analysis that levered external data. These included, for example, the non-synonymous *PTPN22* SNP rs2476601 which was associated with myasthenia gravis (overall and the late-onset subset) by subset-selected FDR < 0.01. This SNP was previously associated with myasthenia gravis in a different study [34], and lack of clear replication in the data analysed here (*p* = 6 × 10^−5^) was attributed to differences in population structure. Eight associations (five variants) were not previously reported to our knowledge, including associations near *IRF1/IL5* for myasthenia gravis, near *TNFSF11* for RF− JIA, and near *CD2/CD2*8 for EGPA.

### Component interpretation

**Fig. 5:**
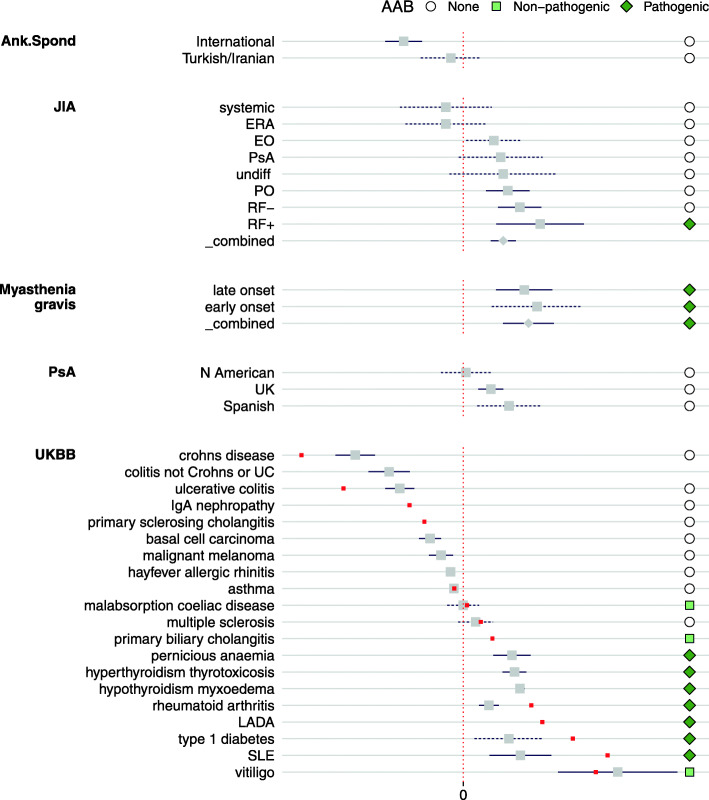
Forest plots showing projected values for diseases significant overall and on components 1. Grey square dots indicate projected data and 95% confidence intervals. Red dots indicate the 13 IMD used for basis construction and for which no confidence interval is available. Points to the right of each line indicate disease classification according to whether they have specific autoantibodies that are either directly implicated in disease pathogenesis (“pathogenic”) or which are specific to the disease, but not involved in pathogenesis (“non-pathogenic”). Diseases that are not associated with specific autoantibodies were classified as “none”. ANCA−, anti-neutrophil cytoplasmic antibody negative; Ank. Spond, ankylosing spondylitis; EGPA, eosinophilic granulomatosis with polyangiitis; EO, extended oligo; ERA, juvenile enthesitis-related arthritis; IgGPos, IgG positive; JIA, juvenile idiopathic arthritis; LADA, latent autoimmune diabetes in adults; NMO, neuromyelitis optica; PO, persistent oligo; PsA, psoriatic arthritis; RF+/−, polyarticular rheumatoid factor positive/negative; SLE, systemic lupus erythematosus; UC, ulcerative colitis

*PC1*, which explained the greatest variation in the training datasets, appears to represent an autoimmune/(auto)inflammatory axis [35], also characterised by whether diseases are considered antibody “seropositive” or “seronegative” (Fig. 5). The exception is vitiligo, in which, despite strong evidence of T cell autoimmunity, autoantibodies are reported but are not consistent features of disease [36]. Weaker but significant association of psoriatic arthritis (PsA) amongst the other seropositive IMD is also consistent with a recent report of novel pathogenic antibodies in PsA [37]. On the inflammatory/seronegative side, we also saw weaker but still significant signals for atopy, basal cell carcinoma, and malignant melanoma. Both malignant melanoma and non-melanoma skin cancer incidence is increased in IBD, but the relative role of treatment or IBD itself in driving this is hard to determine [38, 39]. On the seropositive side, we saw significant results for pernicious anaemia, a disease strongly associated with anti-gastric parietal cell and anti-intrinsic factor antibodies, as well as with autoimmune thyroiditis, T1D, and vitiligo [40].

To help characterise the biology captured by individual components, we projected additional datasets: blood counts [18], immune cell counts [19], and serum cytokine concentrations [20] (Additional File 2: Tables S5, S6, and S7). Testing for consistency identified outliers in the blood count data, which had been generated from a much larger sample, and so we additionally filtered on consistency in that dataset. These data aided interpretation of two further components.

**Fig. 6:**
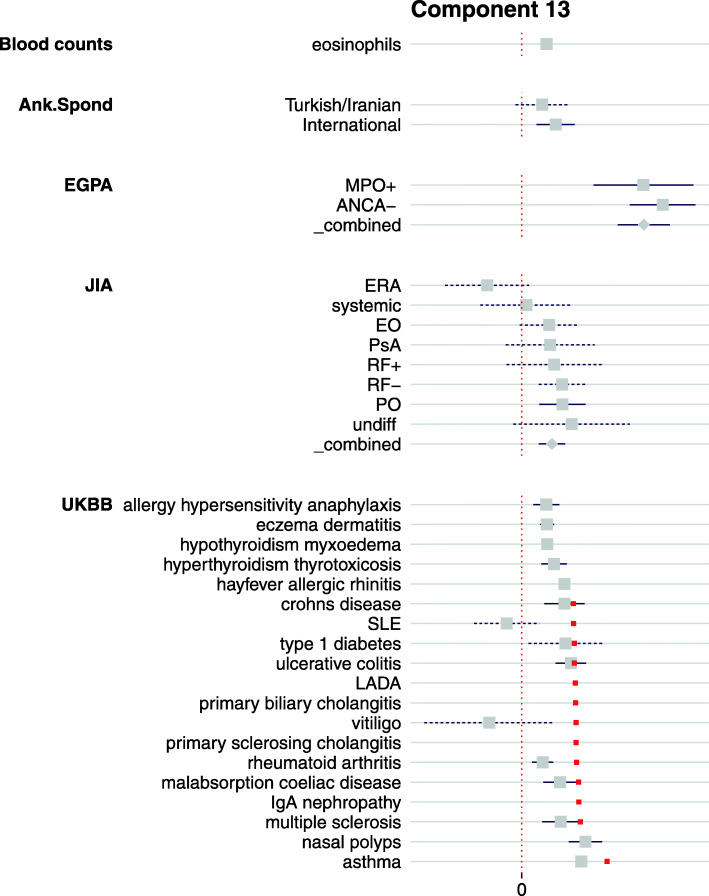
Forest plot of significant traits on PC13 which also shows association with eosinophil counts in blood. ANCA−, anti-neutrophil cytoplasmic antibody negative; Ank. Spond, ankylosing spondylitis; EGPA, eosinophilic granulomatosis with polyangiitis; JIA, juvenile idiopathic arthritis; MPO+, myeloperoxidase positive; PO, persistent oligo; RF−, polyarticular rheumatoid factor negative

*PC13* was striking for the general association of many diseases across all four main clusters in a concordant direction and was the only component for which any projected trait was more extreme than any original basis trait (Fig. 6). The most extreme was EGPA, both ANCA+ and ANCA− subtypes. EGPA is a rare form of AAV (annual incidence 1–2 cases per million) for which genetic differences relating to autoantibody status have been identified [12]. We found PC13 was strongly associated with higher eosinophil counts in a population cohort [18] (FDR < 10^−200^), suggesting that this component describes eosinophilic involvement in IMD. This is consistent with the extreme projection of EGPA which is classified as an eosinophilic form of AAV with both asthma and raised eosinophil count included in its diagnostic criteria.

Eosinophils are pro-inflammatory leukocytes with an established role in atopic diseases such as asthma [41], inflammatory diseases such as IBD [42], and autoimmune diseases such as RA [43]. Mendelian randomisation (MR) analysis of blood cell traits had previously further associated eosinophils with celiac disease (CEL), asthma, and T1D [18]. Our analysis thus supports earlier findings and extends the list of IMD with genetically supported involvement of eosinophils to include EGPA, JIA subtypes, AS, ATD, MS, hay fever, and eczema, in agreement with other recent findings [44].

**Fig. 7:**
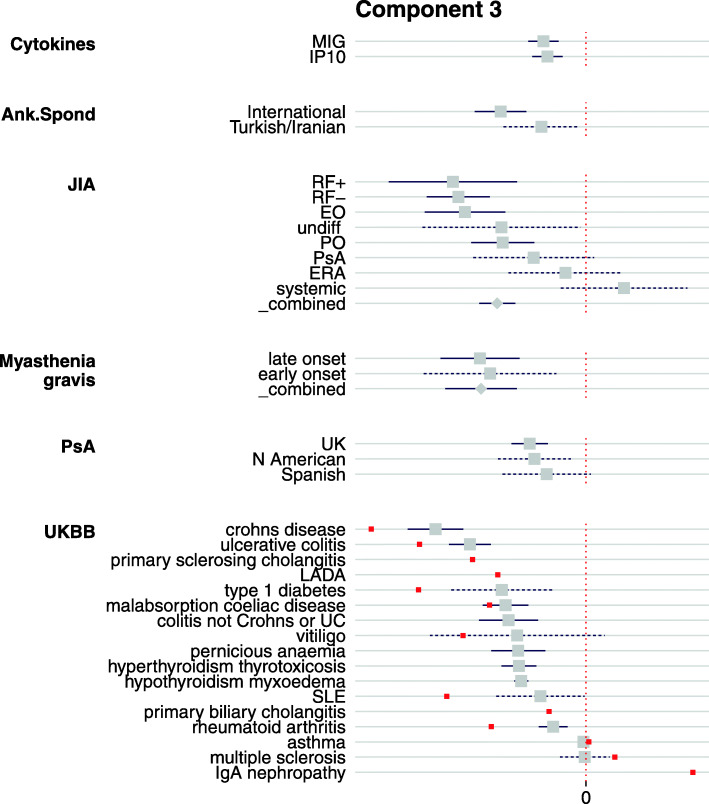
Forest plot of significant traits on PC3 which also shows association with serum cytokine levels of IP-10 (CXCL10) and MIG (CXCL9). EO, extended oligo; PO, persistent oligo; RF+/−, polyarticular rheumatoid factor positive/negative; UC, ulcerative colitis

*PC3* (Fig. 7) was the only component which showed a significant relationship with any serum cytokine concentration. Higher concentrations of CXCL9 (MIG) and CXCL10 (IP-10), Th1 chemoattractants and ligands to the regulator of leukocyte trafficking CXCR3, were both significant in the same direction as several autoimmune diseases, with strongest signals for myasthenia gravis, and several JIA subtypes, as well as IBD, CEL, AS, and sarcoidosis. IP-10 and MIG are chemokines, secreted by epithelial and dendritic cells (amongst others), which act as chemoattractants for immune cells which express the receptor CXCR3, including Th1 cells. Both MIG and IP-10 expression at the site of autoimmune target have been implicated in the development of autoimmunity [45, 46], and IP-10 has been observed to be upregulated in follicular cells of patients with myasthenia gravis [47]. Serum IP-10 has also been found to be raised in patients with recent-onset T1D [48, 49] and Graves’ disease (hyperthyroidism) [46], and to correlate with increased disease activity in SLE [50] and AS [51].

## Discussion

Our motivation in this work was threefold. The first is to overcome the problems of dimensionality and allow an overview of genetic association patterns from multiple related diseases without oversimplification. While previous efforts to relate different traits through GWAS statistics have focused on large studies and shown that they can distinguish broad classes of immune-mediated, cardiovascular, and metabolic diseases [6, 14], we have tackled the problem of finding structure *within* a single class of diseases. Unlike other applications of PCA to genetics, we split our datasets into “training” and “test” sets, enabling standard statistical hypothesis testing and providing robustness against overfitting. Importantly, our method allows synthesis of knowledge from different studies, allowing large numbers of cases from different diseases to contribute to the constructed dimensions.

Our second motivation was to generate new knowledge in rare IMD. The number of polymorphic human genetic variants together with our understanding that genetic effects on human disease are generally modest has led to massive GWAS to overcome the penalty that must be applied for multiple testing. This is simply not possible for rare diseases. One of the tools which has enhanced rare disease GWAS is the borrowing of information from larger GWAS of aetiologically related diseases [12], and our basis serves a similar function here. By leveraging information about a SNP’s potential to be IMD-associated, we can both increase genetic discovery and place less common diseases in the context of their more prevalent counterparts. More generally, studies of SRD are being enabled on a massive scale by UKBB [52] and 23andMe [53], although studies of such cohorts tend to focus on the more common diseases such as type 2 diabetes (T2D) and coronary heart disease. Our results provide reassurance that SRD associations are consistent with those from targeted GWAS, and extend their utility to IMD and other diseases which are generally found at a lower frequency.

Our final motivation was to extract different axes underlying IMD genetic risk. Works in metabolic [54] and psychiatric [55] diseases have attempted to learn composite factors underlying risk of these related diseases through deeper phenotyping of patients before testing these factors for genetic association. Alternatively, decomposition of estimated effects at 94 T2D risk variants, together with their effects on 46 metabolic traits, was used to cluster variants into 5 groups, three focused on insulin resistance and two on beta cell function [56]. Here, we hoped to learn the same sorts of factors by decomposing only summary GWAS data on clinical disease endpoints. Our continuous shrinkage weight learnt across all training datasets enables us to extract disease-relevant structure, with projected traits lying close to their training data counterparts, something achieved with disease-specific hard-thresholded weights [15] for only the largest datasets.

There are limitations with the method. The assumption of a single causal variant per disease, and per LD-defined region, in generating SNP weights is obviously unrealistic. However, it is this simple assumption that allows us to process summary GWAS data from multiple studies without accurate LD estimates from each study. The assumption, while simplistic, has nonetheless been used in both fine-mapping and colocalisation analyses, because in most cases it means only the strongest signal in each region is considered per disease [57]. More sophisticated fine-mapping methods which can cope with multiple causal variants in LD will be required to adapt our method to the MHC which harbours many of the strongest IMD effects. A more impactful limitation is likely to be that signals in projected datasets can only be discovered if they are also captured in the diseases used to build the basis. Thus, the careful selection of plausibly relevant traits is important, and a negative result for a projected dataset only means no detected association with the identified components, and not an absence of genetic association. For example, the relative underrepresentation of atopic diseases in our input datasets may underlie the relative lack of associations seen for allergy and eczema. The number of available input datasets also limits the number of components that may be distinguished to the rank of the matrix of shrunk effect sizes, which cannot be greater than the number of datasets. For both these reasons, future work will seek to expand the number of datasets included to develop a more comprehensive IMD basis.

We found components defined using the largest GWAS of IMD we could access showed different patterns of association with different disease subsets, emphasising the utility of a multidimensional view. The autoimmune/(auto)inflammatory axis in IMD represented by PC1 is well documented, with the gradient along PC1 corresponding to a shift from autoantibody seronegative to seropositive diseases. Significant IMD on the MIG/IP-10-associated PC3 included both “seropositive” and “seronegative” diseases, although not atopy, while all three groups were represented on the eosinophil-associated PC13. While these observations support a link between certain IMD and serum cytokine levels or blood cell counts, our results do not directly implicate these as causal. Both cytokines and blood count data were measured in unselected population cohorts which will include individuals with IMD, such that the association with IMD may be causal or consequential. For example, we can conclude only that PC3 represents an IMD-related process that contributes to serum cytokine levels. Nonetheless, clinical efficacy of MDX1100, a monoclonal antibody to IP-10, has been demonstrated in RA [58] and a dose-response relationship observed in UC [59]. Our results suggest IP-10 blockade might also be considered in patients with myasthenia gravis, JIA, AS, and sarcoidosis.

## Conclusions

Our proposed approach may be considered a form of feature engineering. We represent genetic associations for aetiologically related traits using radically fewer features, with attached estimates of uncertainty. This enabled us to identify clusters of IMD and nominate involvement of IP-10 and eosinophil counts as involved in a wider range of IMD than previously suggested. Such observations provide a rationale for potential therapeutic repurposing opportunities. Beyond these uses, we expect that reduced dimensional representation of multiple genetic association datasets will offer a foundation for other novel cross-disease analyses within and beyond the immune-mediated focus here.

## Supplementary Information

**Additional file 1: Supplementary Note.** Mathematical exposition of basis construction.

**Additional file 2: Tables S1-S8.** Summary of input datasets, sources, and sample sizes.

**Additional file 3: Fig. S1-S21.** Distribution of SNPs across the basis components, reconstruction plot error, hierarchical clustering of basis diseases and their basis counterparts using different thresholds, delta plots for the 13 principal components in the IMD basis, test for consistency across trait groups, comparison of projections across different ancestries, distribution of entries in the rotation matrix for each component in the basis, and QQ plots of *p*-values for driver SNPs on trait-significant components.

**Additional file 4.** Methods for GWAS analysis of individual level datasets: vasculitis, JIA and PsA.

**Additional file 5: Table S9.** Input summary statistics for SNPs needed for basis projection for JIA and PsA. Beta refers to the effect of allele a2 compared to a1. se.beta is the standard error of beta. SNPs are identified by chromosome, position, reference and alternative alleles. (CSV 346 kb)

**Additional file 6: Table S10.** Projection results for each studied trait, giving the delta value for each PC, its variance, and the Benjamini-Hochberg adjusted *p* value (“fdr.delta”) together with an overall test of significance, both raw (“p.overall”) and Benjamini-Hochberg adjusted (“fdr.overall”). (CSV 1552 kb)

## Data Availability

All data generated or analysed during this study are included in this published article and its additional information files: Input datasets and sources are summarised in Additional File 2, Tables S1-S8. Input summary for datasets not yet publicly available (JIA and PsA) is given in Additional File 5, Table S9. Projection results for each studied trait are given in Additional File 6, Table S10. An R implementation of the method is available from https://github.com/ollyburren/cupcake/ [62]. Code to run the analyses presented here is available from https://zenodo.org/record/4069214 [63]. We also created an online tool to allow other researchers to project their own data into the basis https://grealesm.shinyapps.io/IMDbasisApp/ [64]. Code underlying this tool is available at https://github.com/GRealesM/IMDbasisApp [65].

## Abbreviations

AAV: ANCA-associated vasculitis
ANCA: Anti-neutrophil cytoplasmic antibody
AS: Ankylosing spondylitis
CEL: Celiac disease
EGPA: Eosinophilic granulomatosis with polyangiitis
ERA: Enthesitis-related arthritis
GWAS: Genome-wide association studies
IBD: Inflammatory bowel disease
IMD: Immune-mediated diseases
JIA: Juvenile idiopathic arthritis
EO: Extended oligoarticular
PO: Persistent oligoarticular
RF+/RF−: Polyarticular rheumatoid factor positive/negative, respectively
LD: Linkage disequilibrium
MAF: Minor allele frequency
MHC: Major histocompatibility complex
MPO: Myeloperoxidase
MS: Multiple sclerosis
NMO: Neuromyelitis optica
PCA: Principal component analysis
PsA: Psoriatic arthritis
SRD: Self-reported diseases
T1D: Type 1 diabetes
T2D: Type 2 diabetes
UC: Ulcerative colitis
