# Supplementary material for: Genetic feature engineering enables characterisation of shared risk factors in immune-mediated diseases
Source: Genome Med. 2020 Nov 25;12:106. doi: 10.1186/s13073-020-00797-4 (PMC7687775; doi:10.1186/s13073-020-00797-4)
Supplement: Supplementary file 3 — Additional file 3: Fig. S1-S21. Distribution of SNPs across the basis components, reconstruction plot error, hierarchical clustering of basis diseases and their basis counterparts using different thresholds, delta plots for the 13 principal components in the IMD basis, test for consistency across trait groups, comparison of projections across different ancestries, distribution of entries in the rotation matrix for each component in the basis, and QQ plots of p-values for driver SNPs on trait-significant components. [file 13073_2020_797_MOESM3_ESM.pdf]

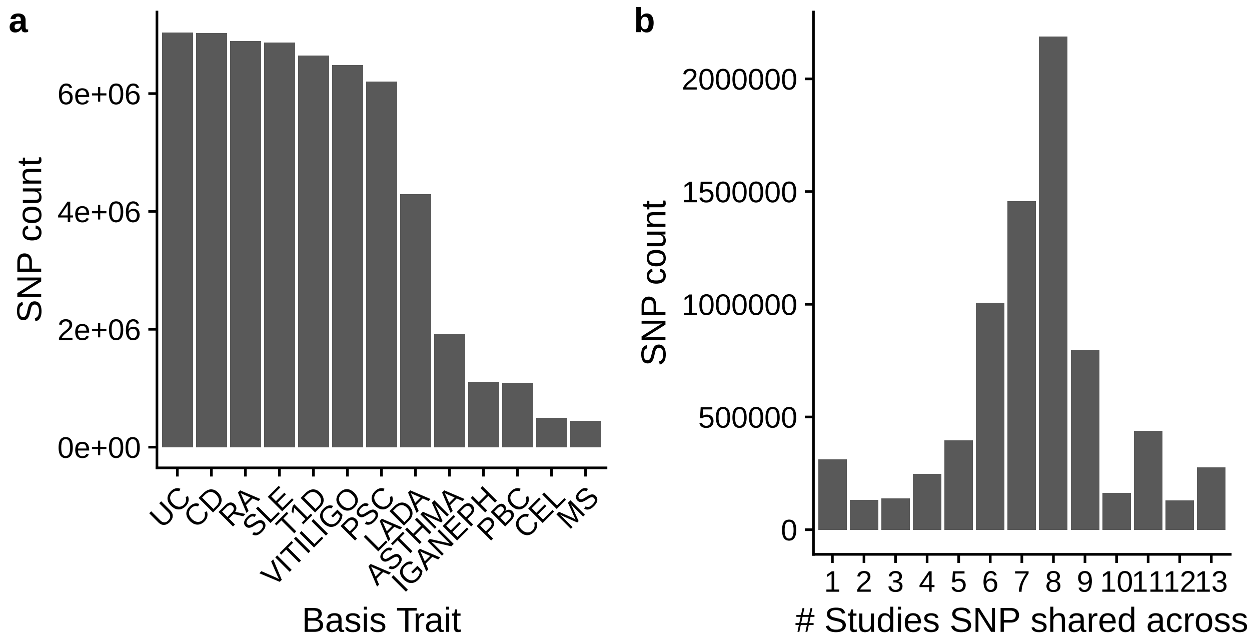

Supplementary Figure 1: SNP coverage across basis traits. **a** number of SNPs available across each basis study. **b** number of SNPs in common across varying numbers of basis traits.

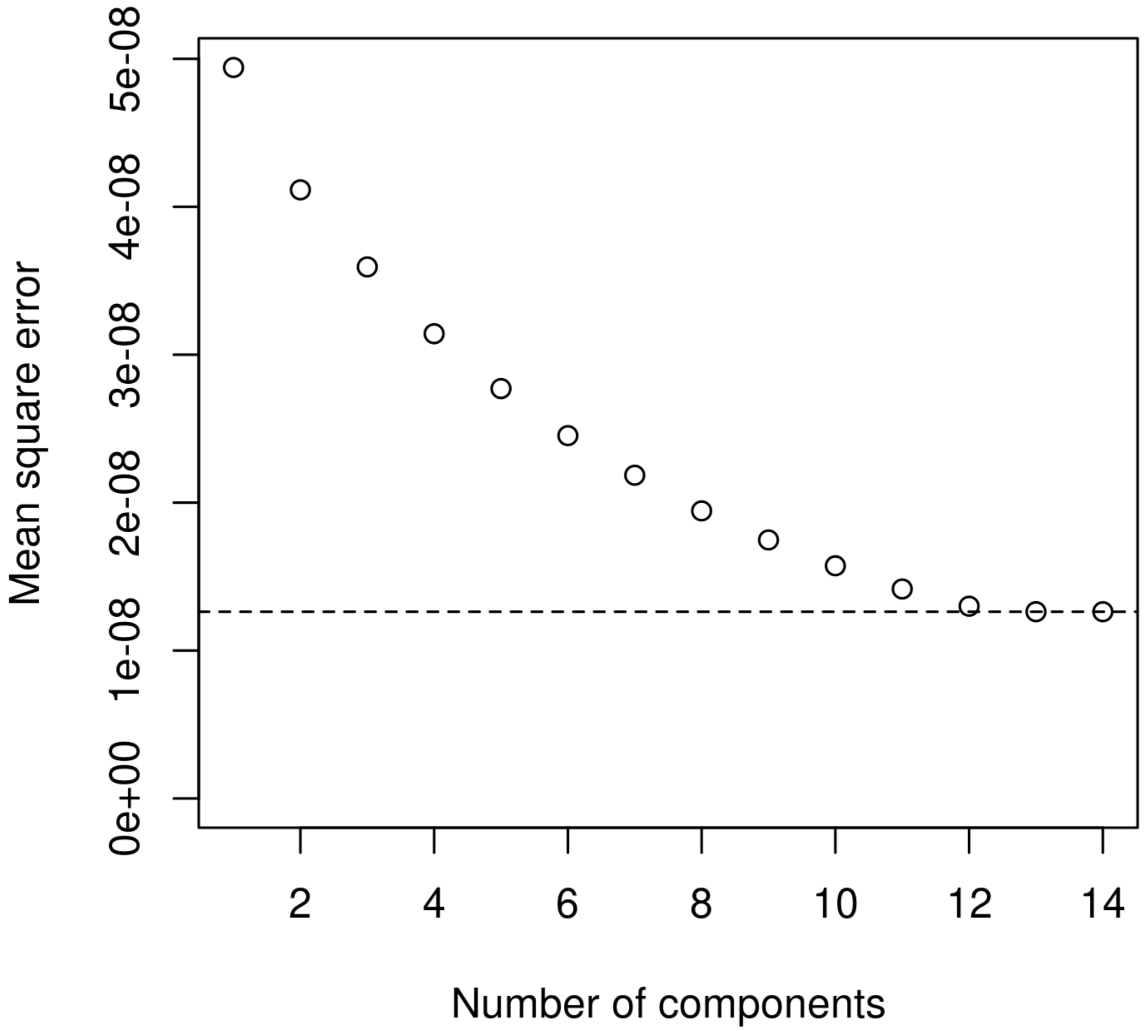

Supplementary Figure 2: Mean squared reconstruction error as the number of components used from the principal component decomposition increases from 1 to 14. The error is minimised with 13 or 14 components.

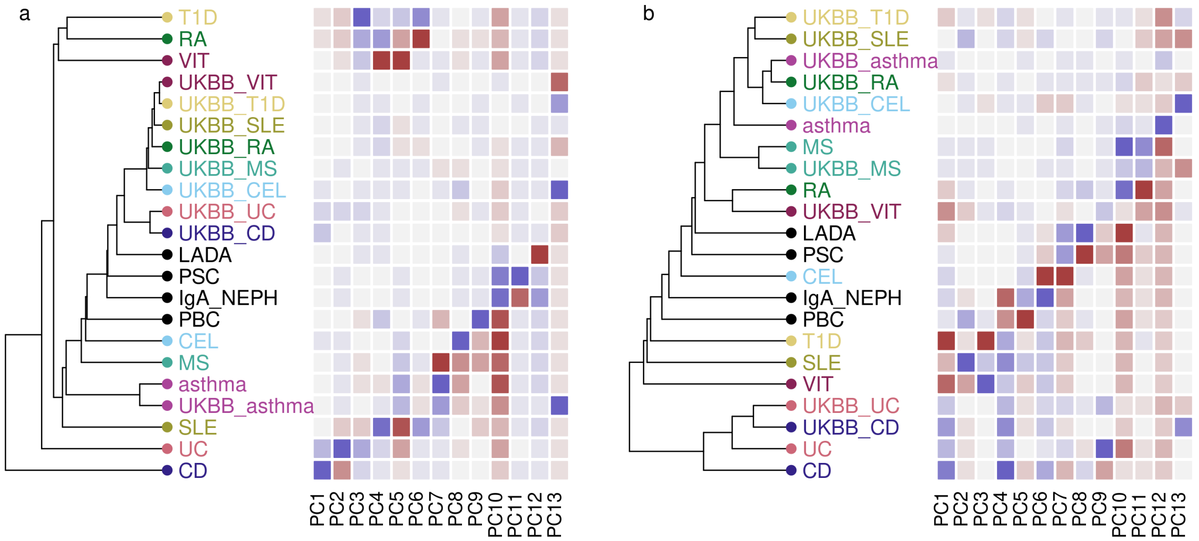

Supplementary Figure 3: Hierarchical clustering of basis diseases and their UKBB counterparts in **a** hard-thresholded, LD-thinned basis constructed using Z scores **b** hard-thresholded, LD-thinned basis constructed using $\hat{\beta}$. Heatmaps indicate projected $\hat{\delta}$ for each disease on each component PC1-PC13, with grey indicating 0 (no difference from control), and darker shades of blue or magenta showing departure from controls in one direction or the other. GWAS datasets: T1D = type 1 diabetes, CEL= celiac disease, asthma, MS =multiple sclerosis, UC =ulcerative colitis, CD = Crohn's disease, RA =rheumatoid arthritis, VIT =vitiligo, SLE =systemic lupus erythematosus, PSC=primary sclerosing cholangitis, PBC=primary biliary cholangitis, LADA=latent autoimmune diabetes in adults, IgA_NEPH= IgA nephropathy. UKBB_ prefixed diseases correspond to self reported disease status in UK Biobank.

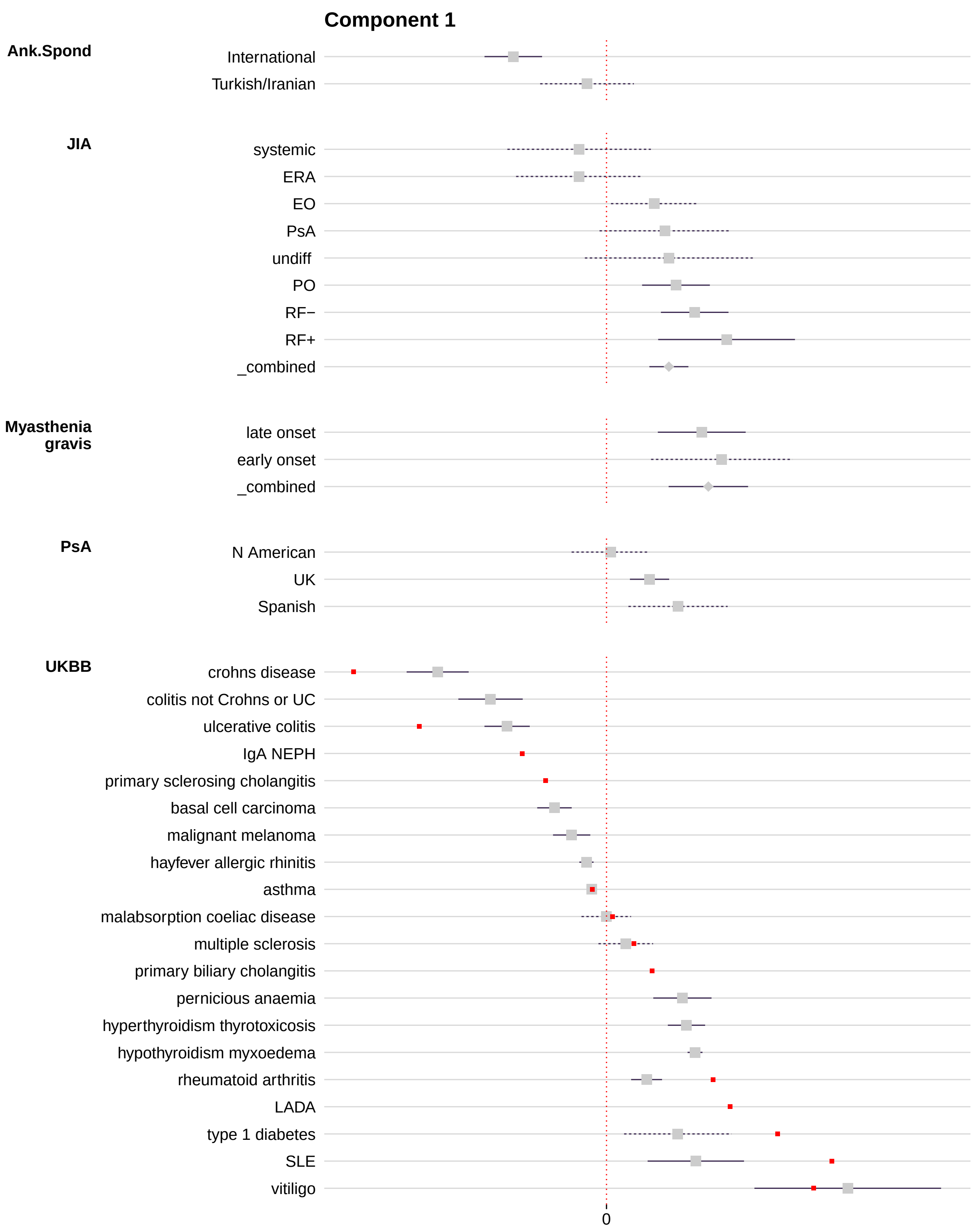

Supplementary Figure 4: Forest plot of component PC1 showing projected delta and 95% confidence interval (solid line = FDR $<$ 1%, dashed line = FDR $\geq$ 1%). All IMD that are part of a trait group with at least one result significant at FDR < 1% are shown, together with any UKBB significant traits. IMD basis disease locations are shown in red.

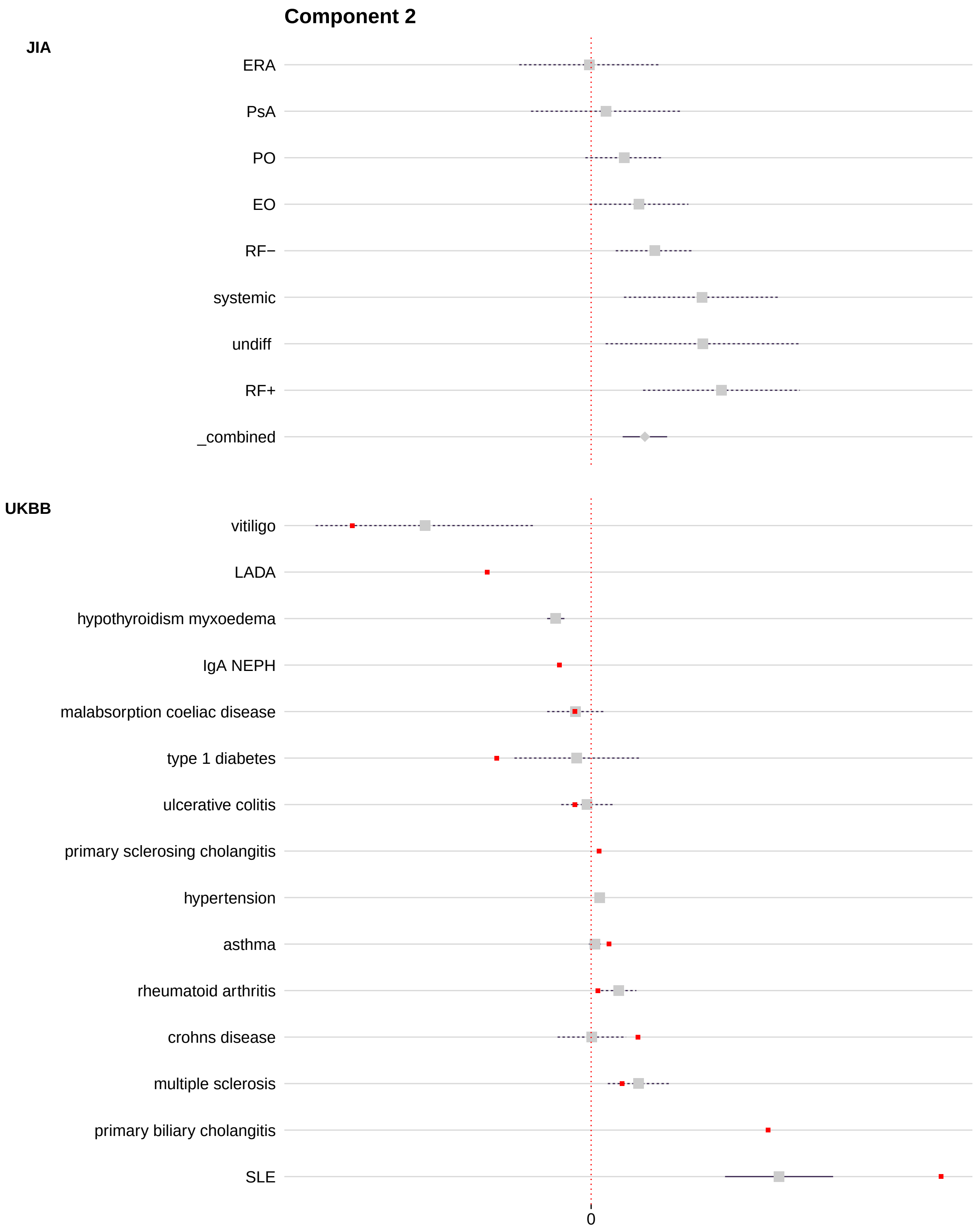

Supplementary Figure 5: Forest plot of component PC2 showing projected delta and 95% confidence interval (solid line = FDR $<$ 1%, dashed line = FDR $\geq$ 1%). All IMD that are part of a trait group with at least one result significant at FDR < 1% are shown, together with any UKBB significant traits. IMD basis disease locations are shown in red.

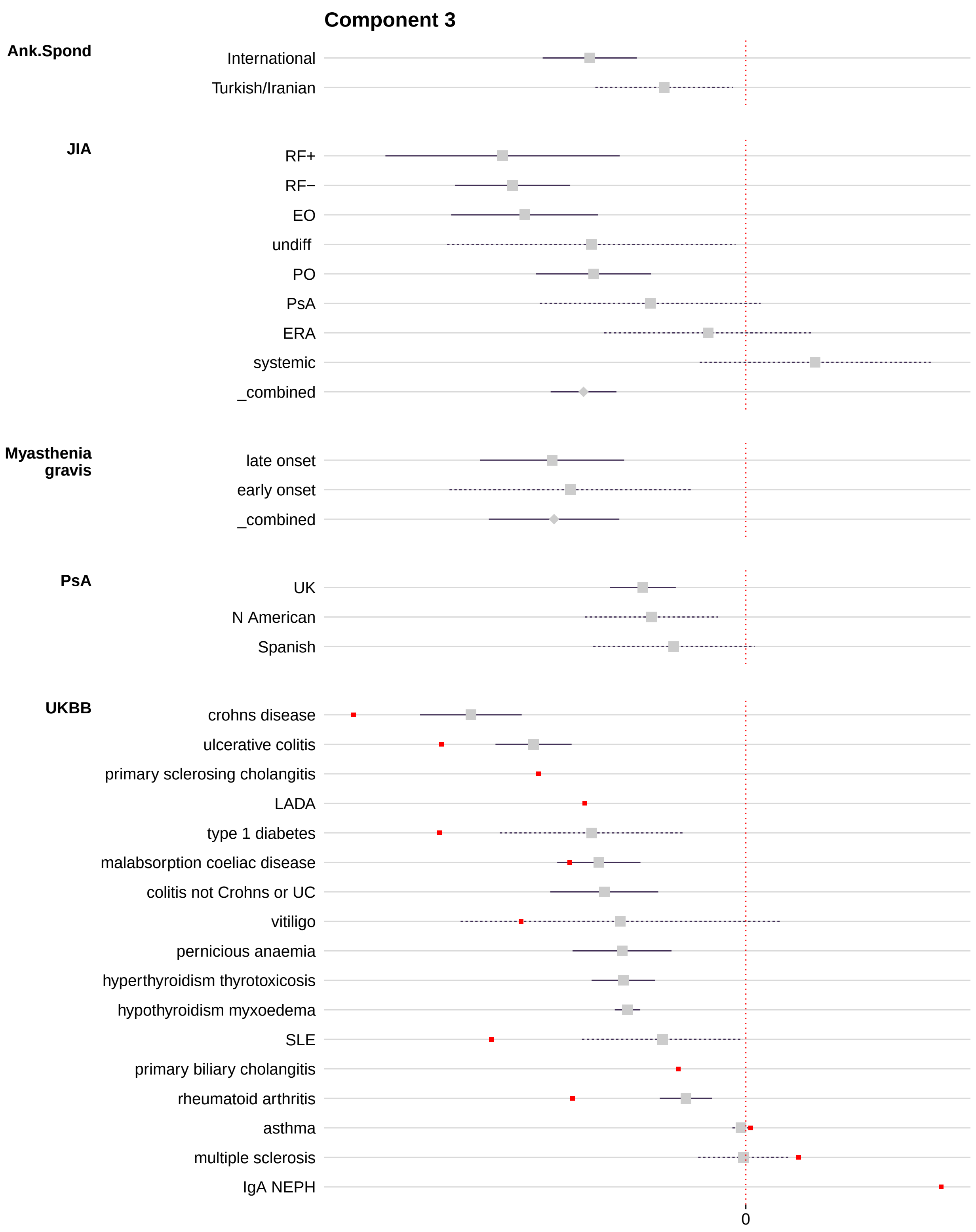

Supplementary Figure 6: Forest plot of component PC3 showing projected delta and 95% confidence interval (solid line = FDR $<$ 1%, dashed line = FDR $\geq$ 1%). All IMD that are part of a trait group with at least one result significant at FDR < 1% are shown, together with any UKBB significant traits. IMD basis disease locations are shown in red.

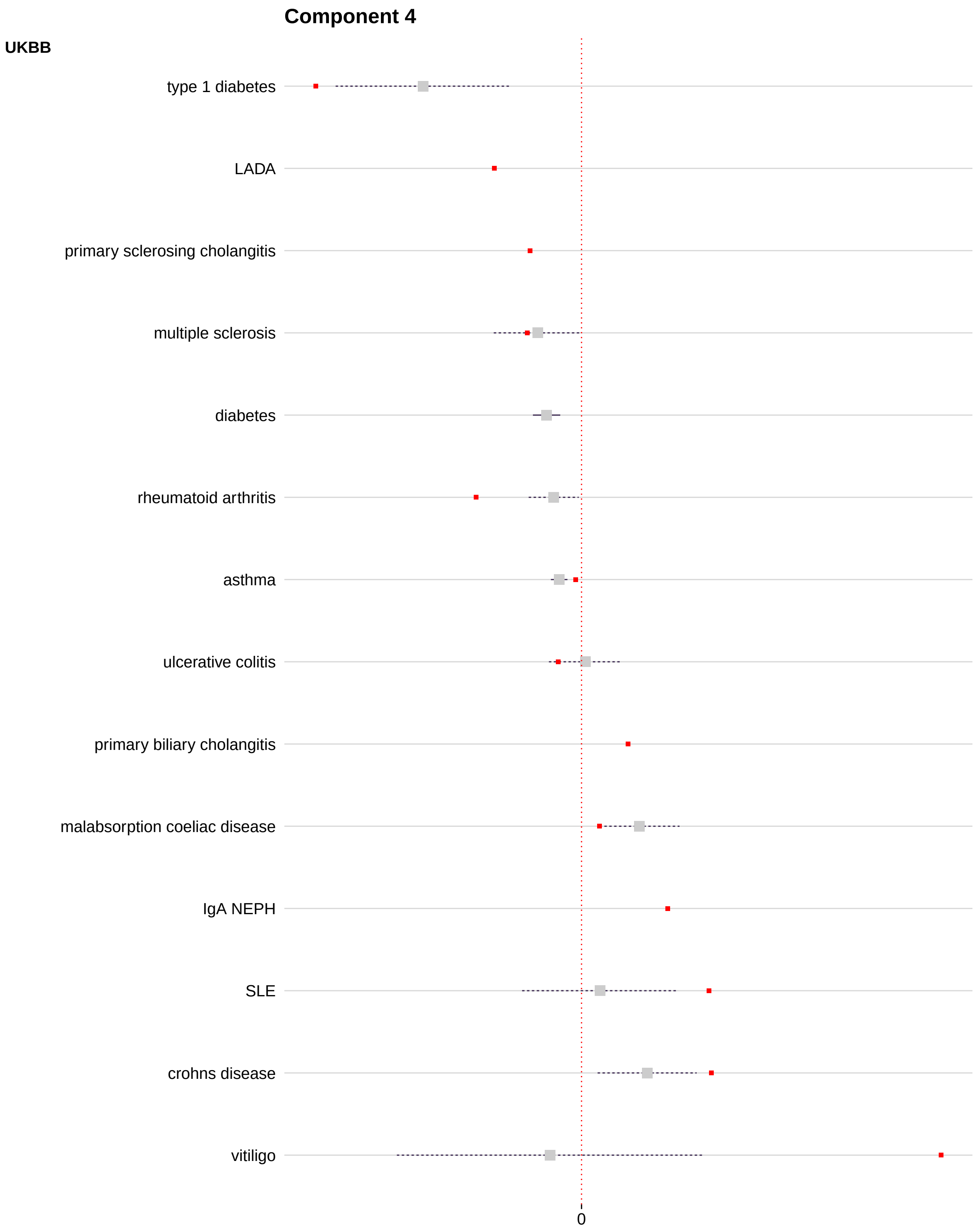

Supplementary Figure 7: Forest plot of component PC4 showing projected delta and 95% confidence interval (solid line = FDR $<$ 1%, dashed line = FDR $\geq$ 1%). All IMD that are part of a trait group with at least one result significant at FDR < 1% are shown, together with any UKBB significant traits. IMD basis disease locations are shown in red.

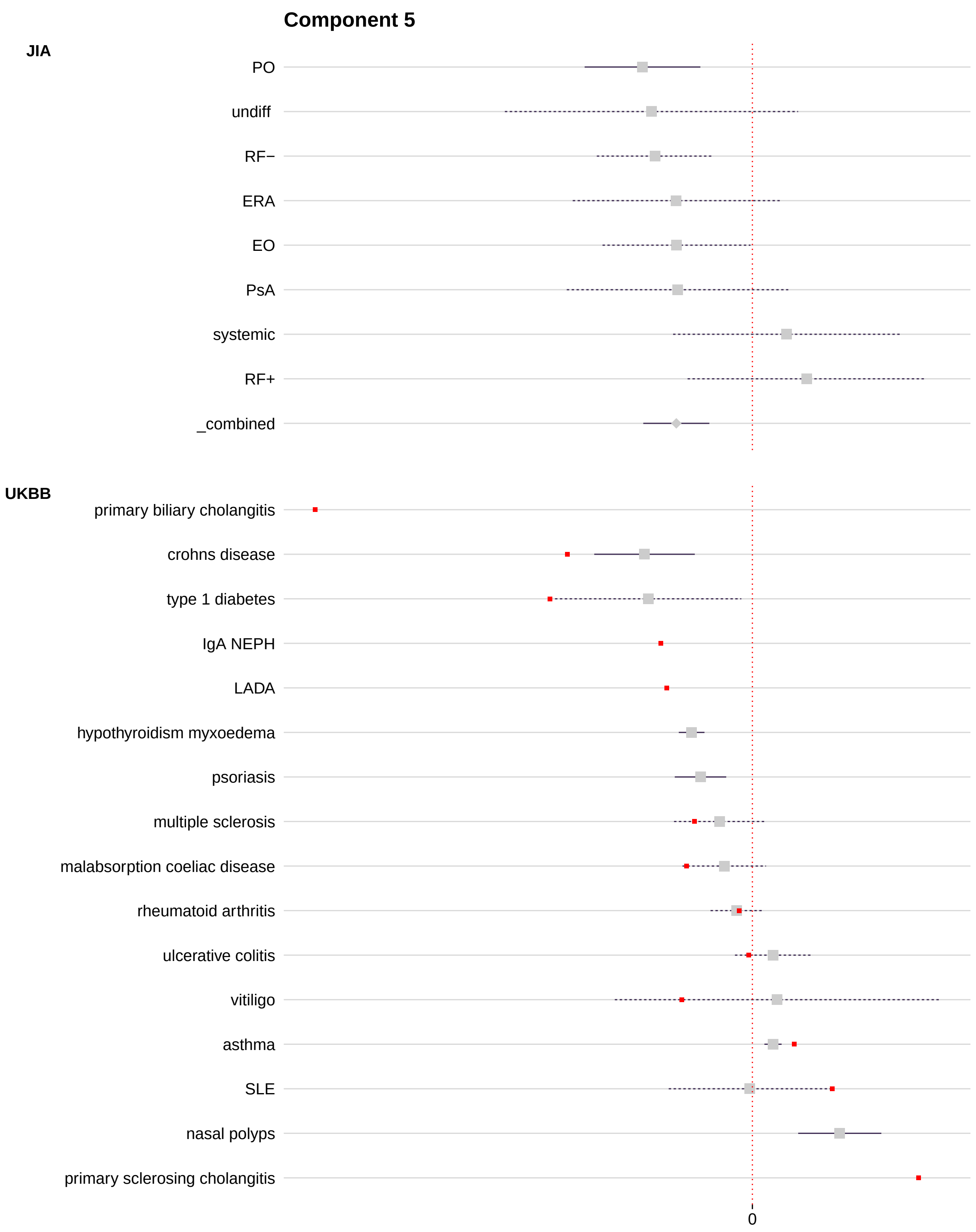

Supplementary Figure 8: Forest plot of component PC5 showing projected delta and 95% confidence interval (solid line = FDR $<$ 1%, dashed line = FDR $\geq$ 1%). All IMD that are part of a trait group with at least one result significant at FDR < 1% are shown, together with any UKBB significant traits. IMD basis disease locations are shown in red.

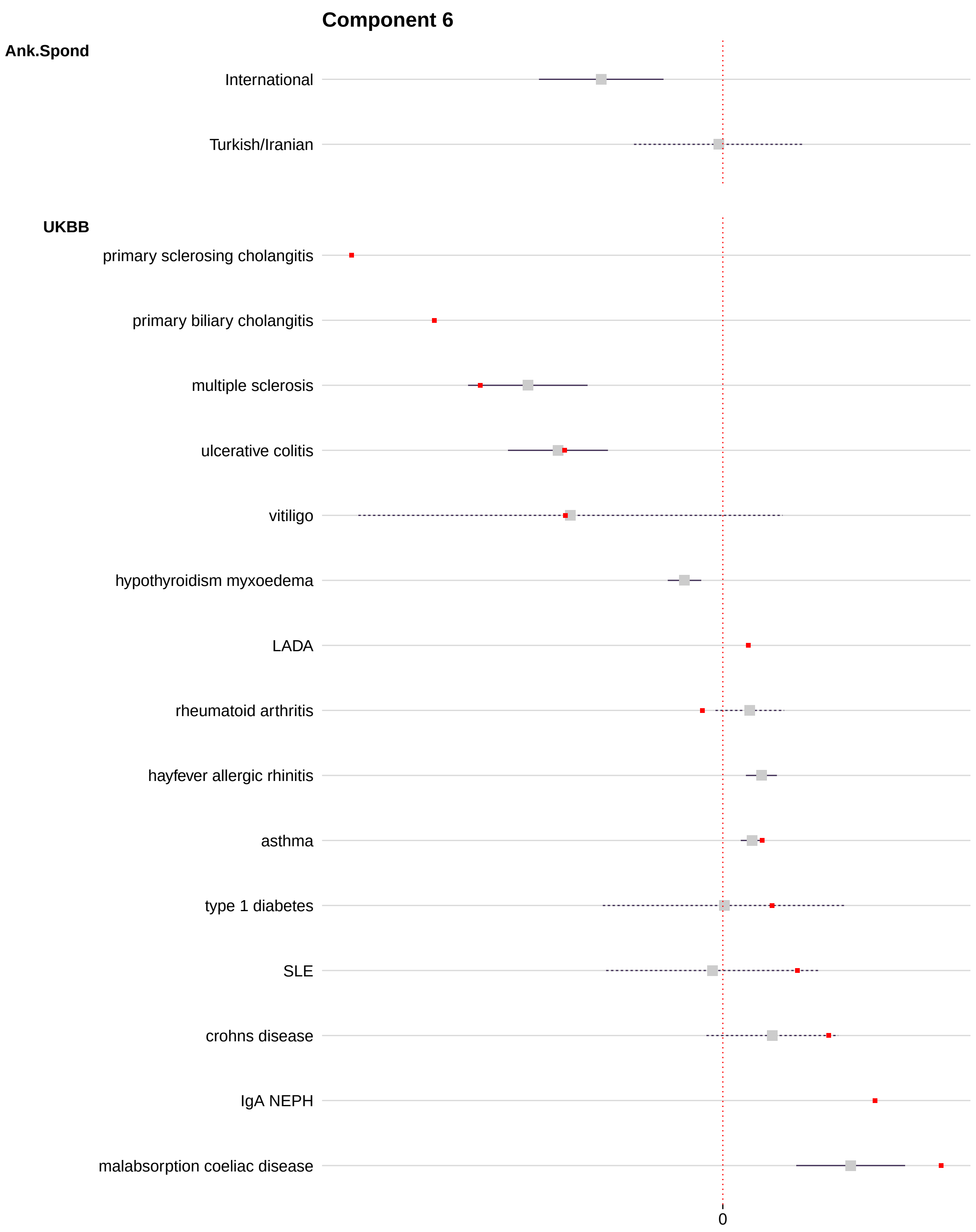

Supplementary Figure 9: Forest plot of component PC6 showing projected delta and 95% confidence interval (solid line = FDR $<$ 1%, dashed line = FDR $\geq$ 1%). All IMD that are part of a trait group with at least one result significant at FDR < 1% are shown, together with any UKBB significant traits. IMD basis disease locations are shown in red.

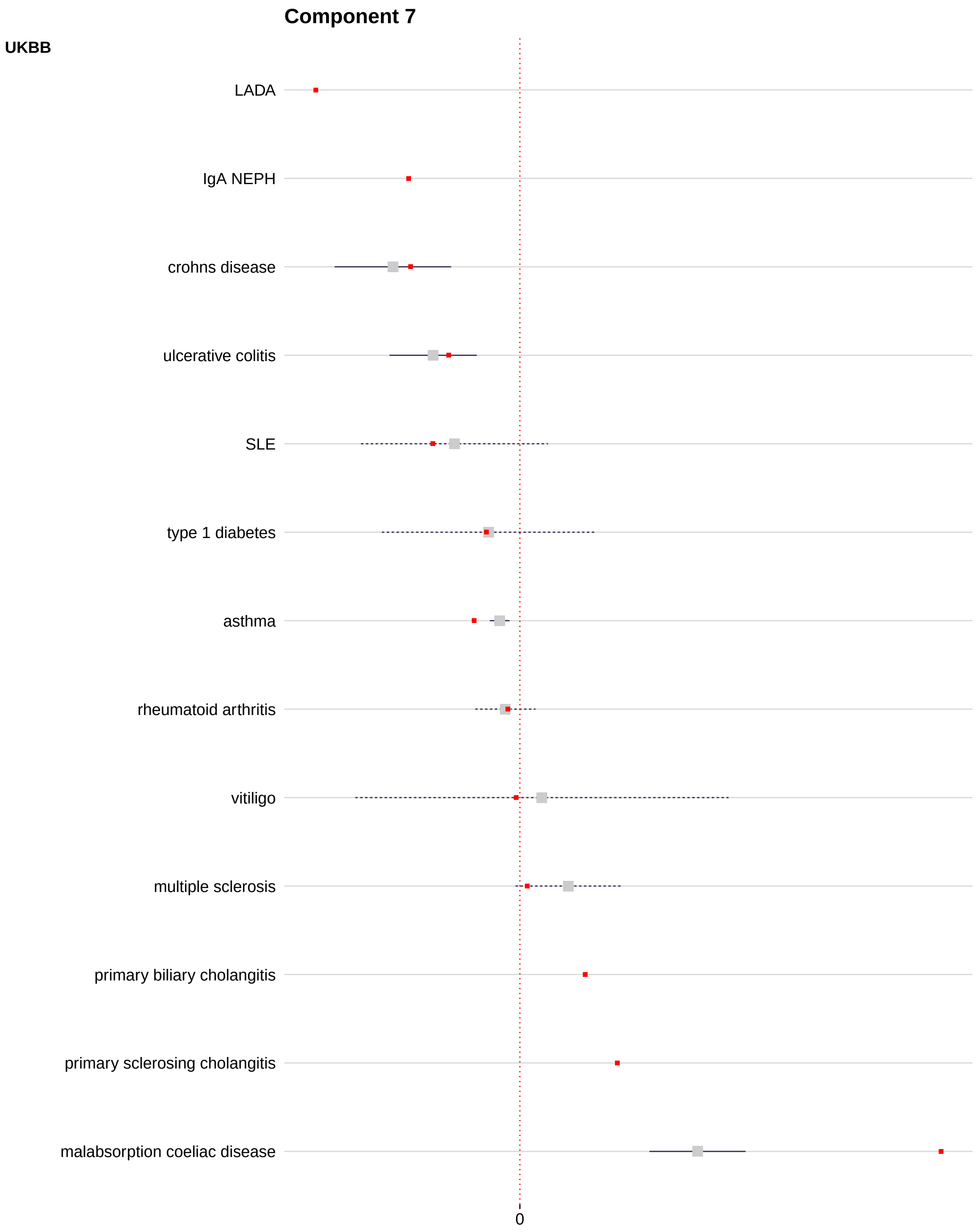

Supplementary Figure 10: Forest plot of component PC7 showing projected delta and 95% confidence interval (solid line = FDR $< 1\%$, dashed line = FDR $\geq 1\%$). All IMD that are part of a trait group with at least one result significant at FDR $< 1\%$ are shown, together with any UKBB significant traits. IMD basis disease locations are shown in red.

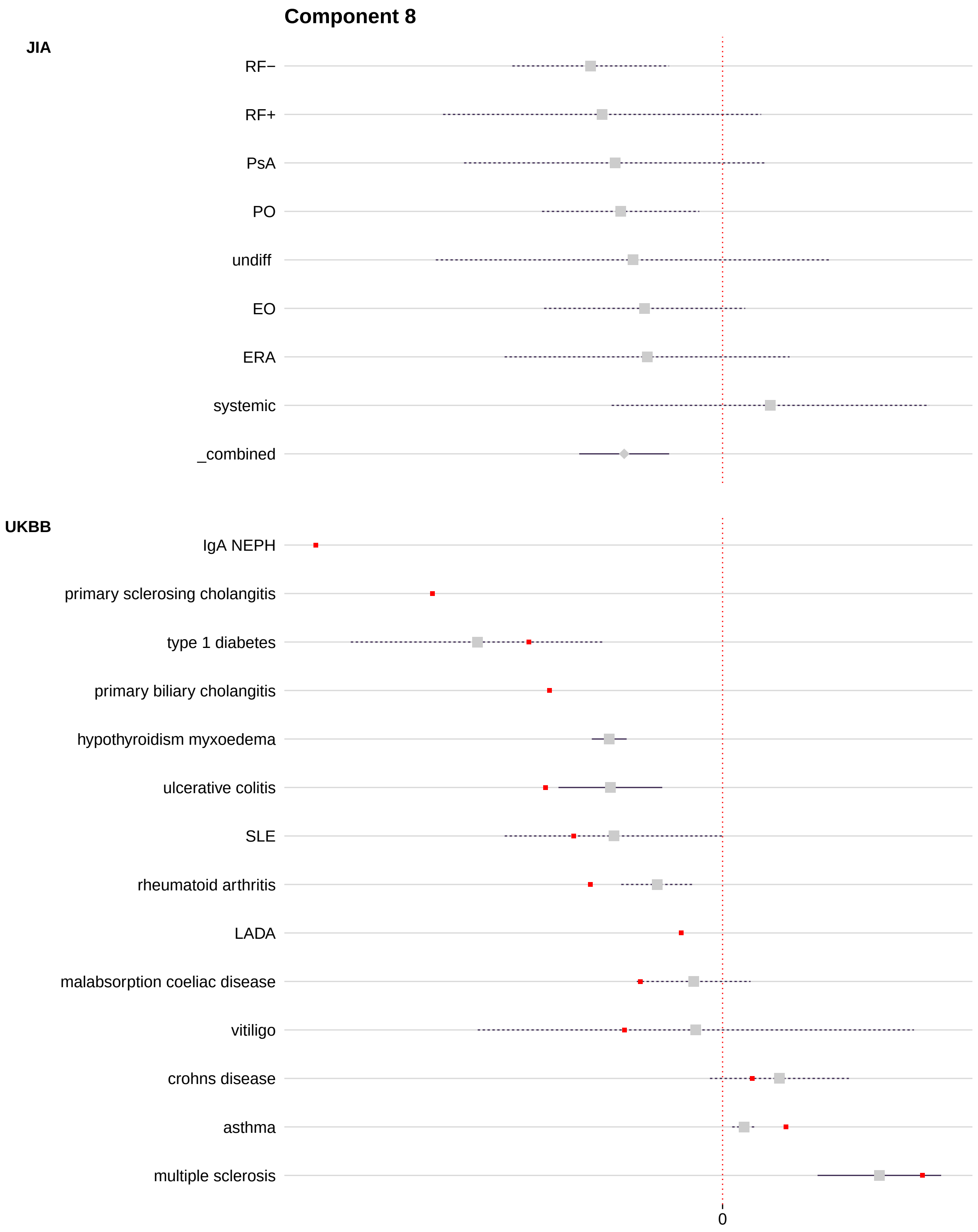

Supplementary Figure 11: Forest plot of component PC8 showing projected delta and 95% confidence interval (solid line = FDR $<$ 1%, dashed line = FDR $\geq$ 1%). All IMD that are part of a trait group with at least one result significant at FDR < 1% are shown, together with any UKBB significant traits. IMD basis disease locations are shown in red.

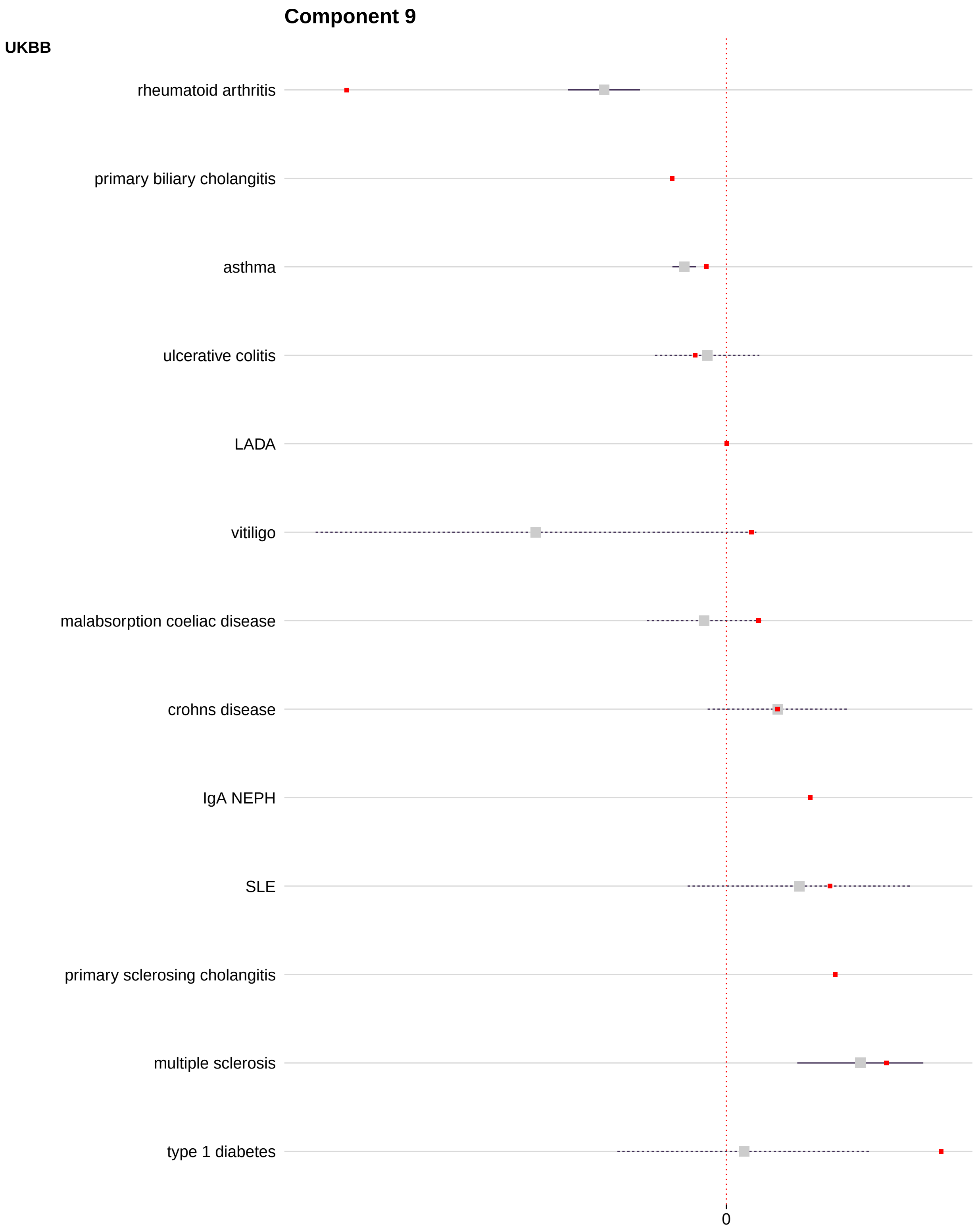

Supplementary Figure 12: Forest plot of component PC9 showing projected delta and 95% confidence interval (solid line = FDR $<$ 1%, dashed line = FDR $\geq$ 1%). All IMD that are part of a trait group with at least one result significant at FDR < 1% are shown, together with any UKBB significant traits. IMD basis disease locations are shown in red.

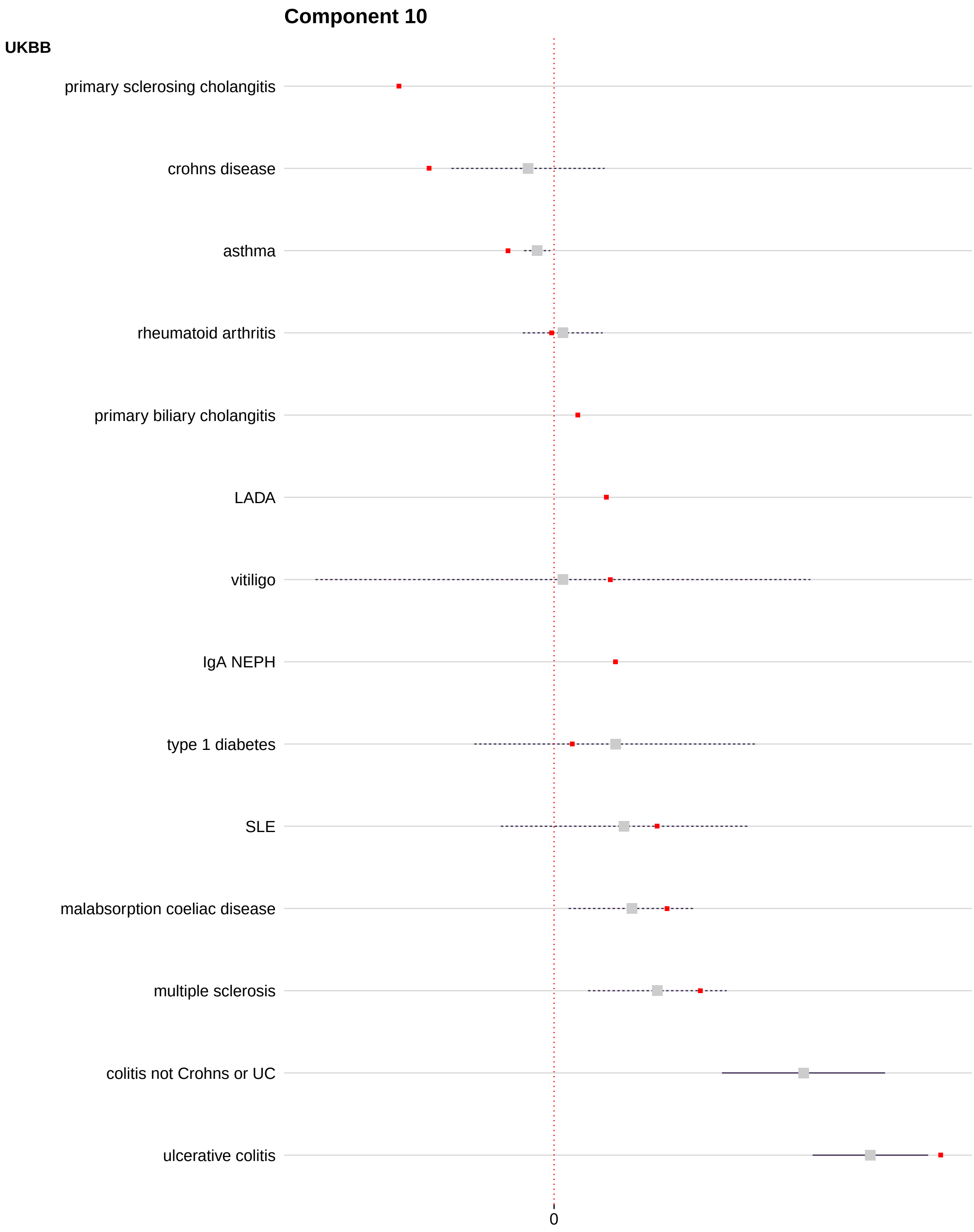

Supplementary Figure 13: Forest plot of component PC10 showing projected delta and 95% confidence interval (solid line = FDR $< 1\%$, dashed line = FDR $\geq 1\%$). All IMD that are part of a trait group with at least one result significant at FDR $< 1\%$ are shown, together with any UKBB significant traits. IMD basis disease locations are shown in red.

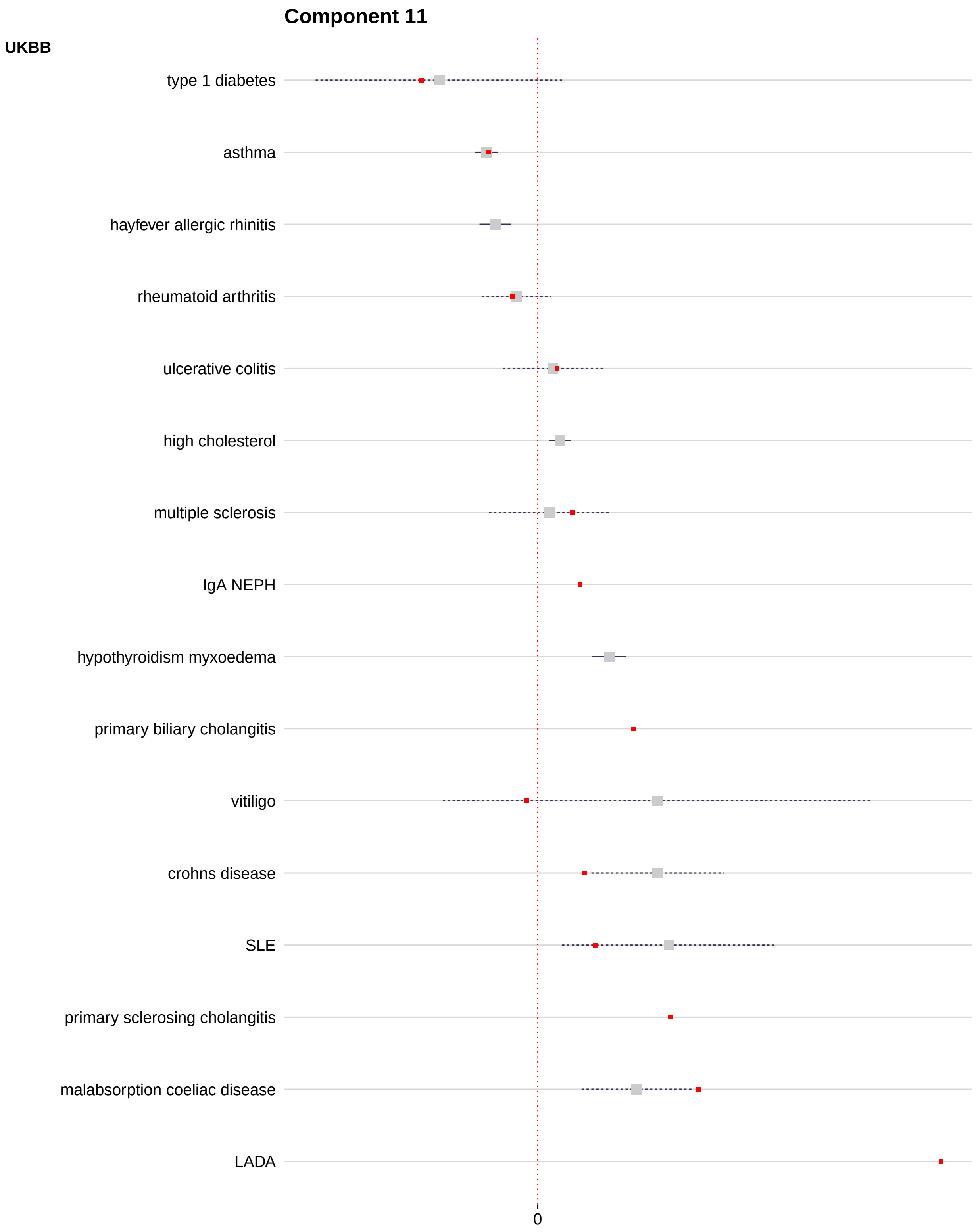

Supplementary Figure 14: Forest plot of component PC11 showing projected delta and 95% confidence interval (solid line = FDR $<$ 1%, dashed line = FDR $\geq$ 1%). All IMD that are part of a trait group with at least one result significant at FDR < 1% are shown, together with any UKBB significant traits. IMD basis disease locations are shown in red.

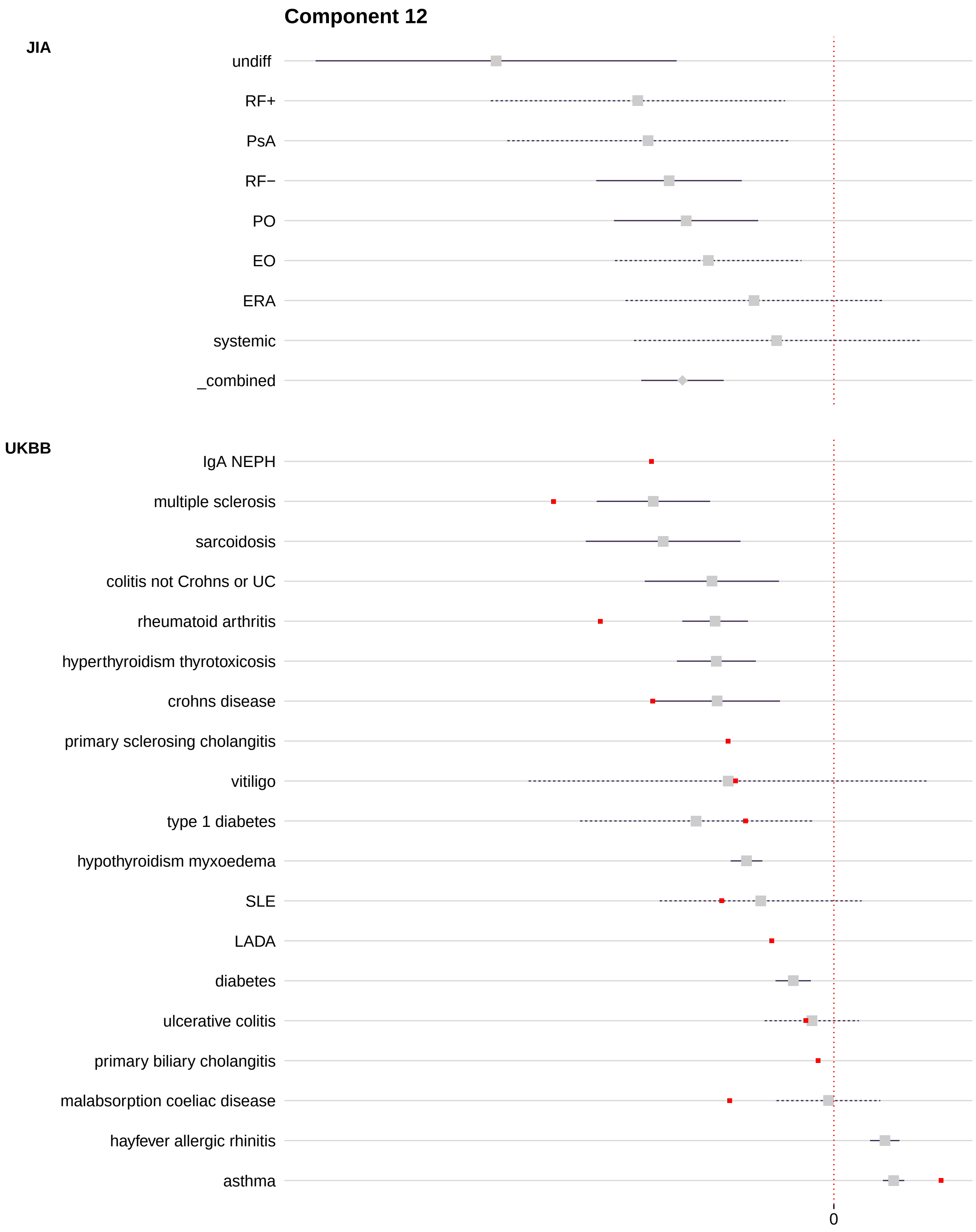

Supplementary Figure 15: Forest plot of component PC12 showing projected delta and 95% confidence interval (solid line = FDR $<$ 1%, dashed line = FDR $\geq$ 1%). All IMD that are part of a trait group with at least one result significant at FDR < 1% are shown, together with any UKBB significant traits. IMD basis disease locations are shown in red.

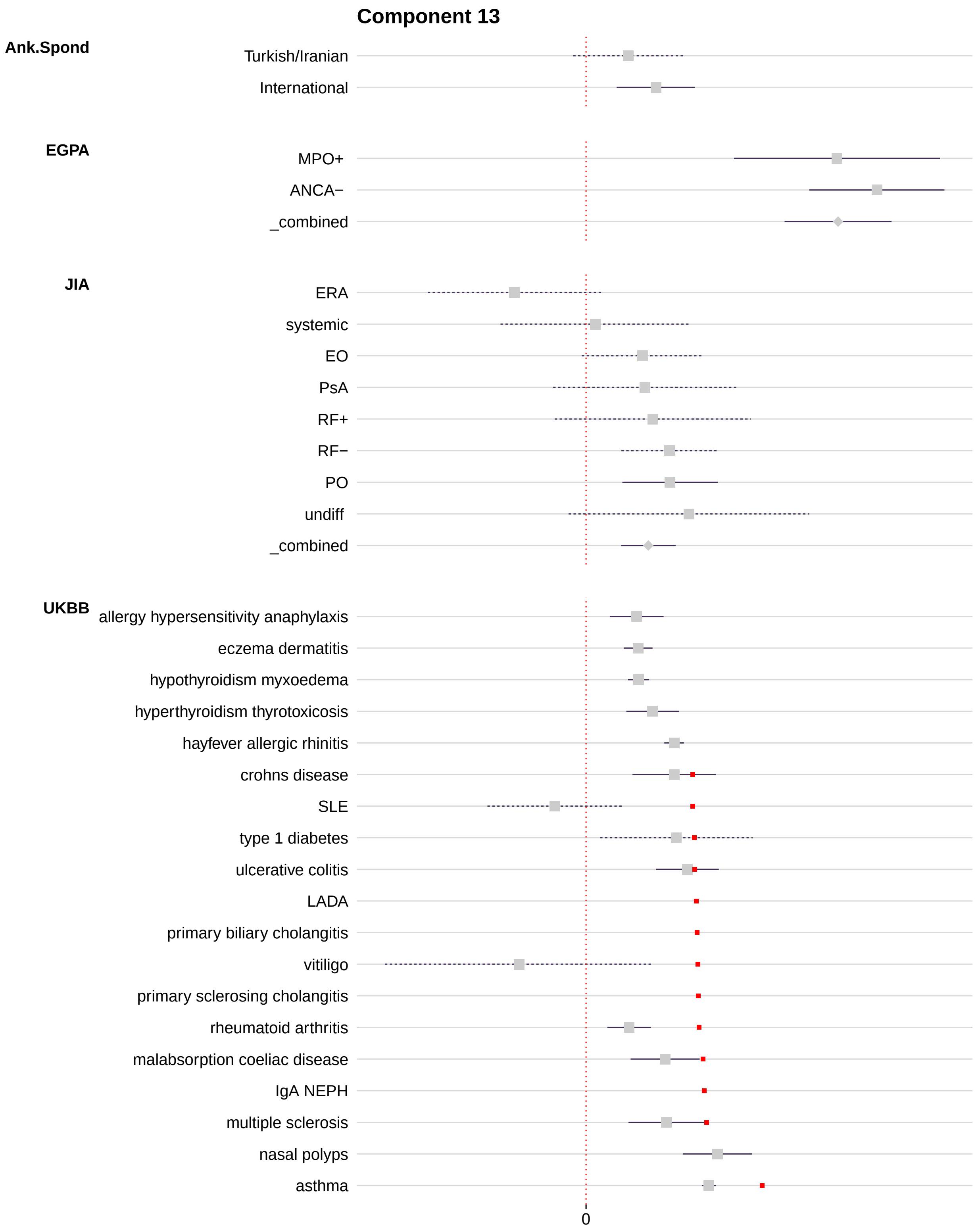

Supplementary Figure 16: Forest plot of component PC13 showing projected delta and 95% confidence interval (solid line = FDR $<$ 1%, dashed line = FDR $\geq$ 1%). All IMD that are part of a trait group with at least one result significant at FDR < 1% are shown, together with any UKBB significant traits. IMD basis disease locations are shown in red.

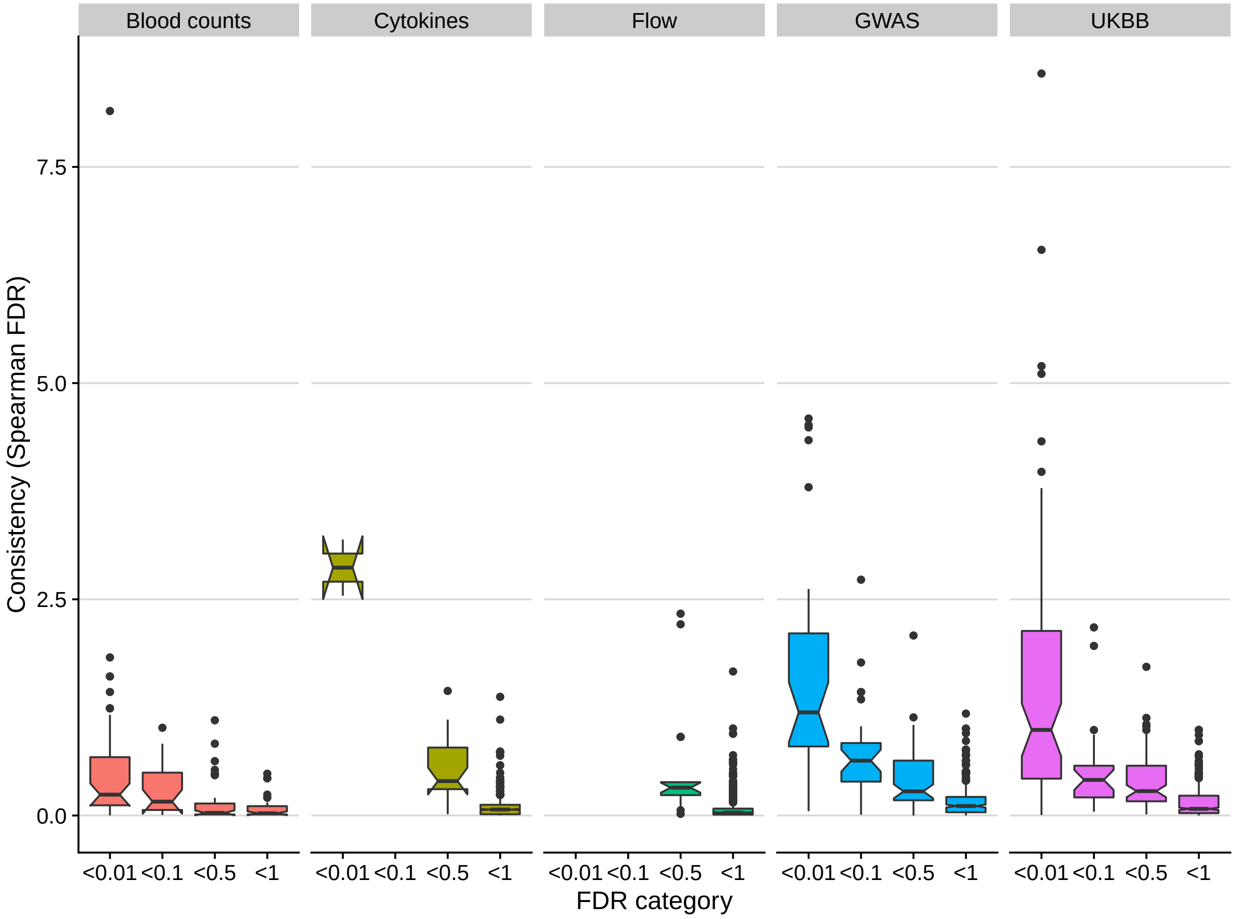

Supplementary Figure 17: For each group of traits, we compared the significance of Spearman correlation test of basis component SNP weights with trait β (evidence for consistency) with component FDR. We found traits with increasing component significance (smaller FDR) tended to also show more significant Spearman correlations, although the pattern was much weaker for blood cell counts despite more observations with small component FDRs. We subsequently filtered blood cell counts to count as significant only the outlying trait which was clearly significant by both component FDR and Spearman correlation, corresponding to eosinophil counts on PC13. Datasets are grouped by: blood cell counts,[21] cytokines,[23] flow cytometric immune cell counts,[22] GWAS datasets except UKBB, UKBB (Neale).

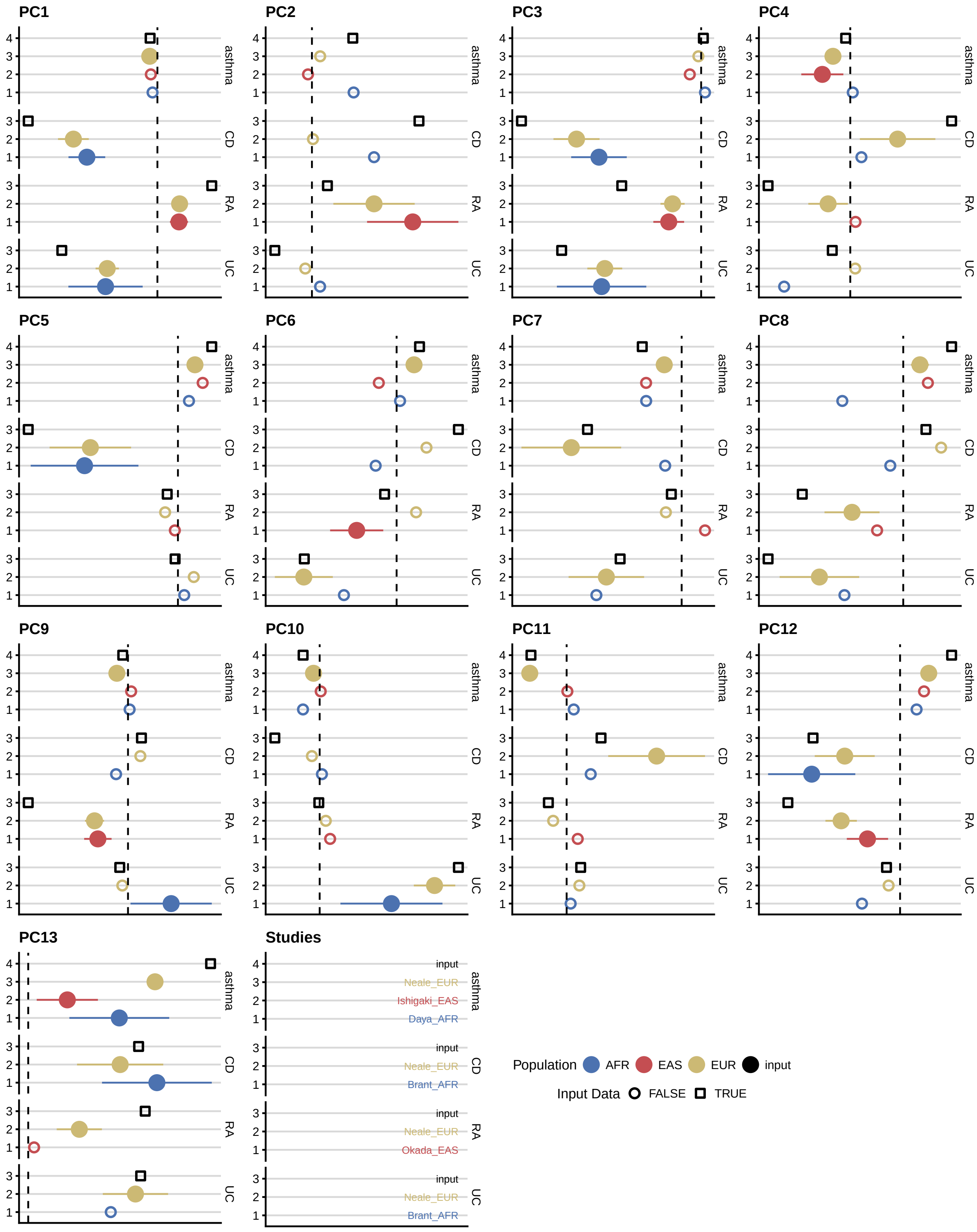

Supplementary Figure 18: Traits with GWAS data from different ancestries were identified, and each dataset projected onto the basis. The location of each dataset (delta) is shown for each PC, coloured by reported ancestry. Solid points had P<0.05 (uncorrected for multiple testing) and lines show their 95% confidence intervals. Hollow points had P > 0.05. The square symbol indicates the location of the input data used to learn the basis, and is shown for reference. The dashed vertical line represents the null hypothesis, delta=0. We find that all significant points have the same sign of delta for any given ancestry and PC combination. AFR=African/African American, EAS=East Asian, EUR=European.

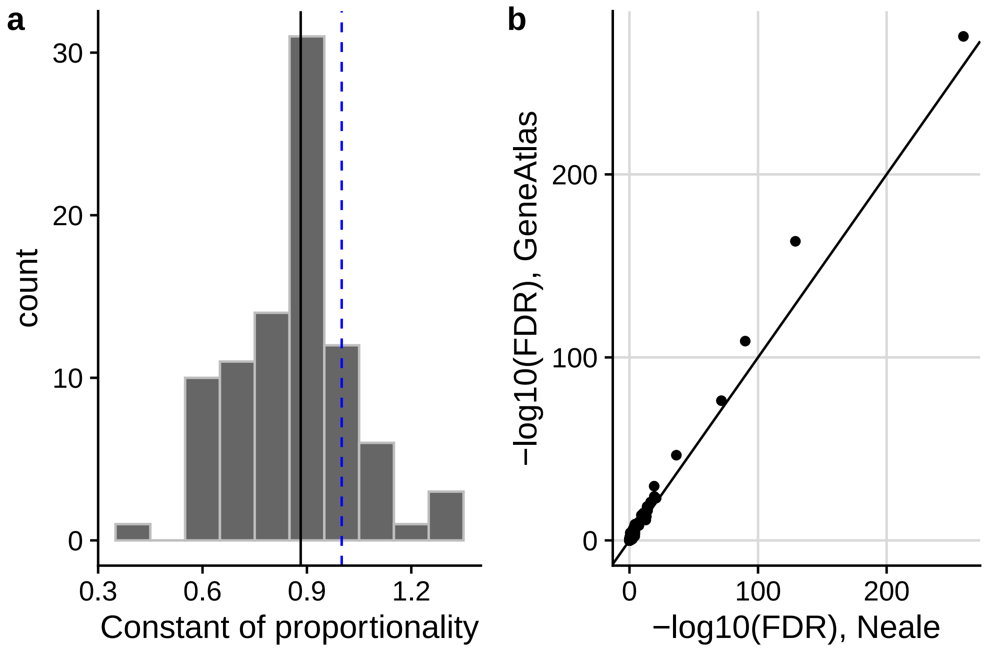

Supplementary Figure 19: Comparison of projects of the same traits from datasets with different ancestries. We ran parallel analyses of two releases of UKBB summary statistics. The Neale compendium focuses on the European subset of UKBB (n approx = 360,000) and GeneAtlas (http://geneatlas.roslin.ed.ac.uk/)(Canela-Xandri, Rawlik, and Tenesa 2018) uses all available UKBB subjects (n approx= 452,000), and a linear mixed model to adjust for population stratification. We tested whether projections for the same trait was proportional across the PCs, and estimated the constant of poportionality for each. Over 78 traits found in both datasets with similar case counts (within 10%), (a) GeneAtlas projections were generally proportional to Neale (no significant deviation from proportionality identified) but attenuated (median ratio=0.89) and (b) the additional sample size in GeneAtlas results in generally more significant projections. This suggests the mix of non-European samples leads to an attenuation of signal compared to European-only. Overall, this suggests that results projecting non-European or GWAS studies on to a European basis may reduce power for the same sample size, but does not lead to invalid results.

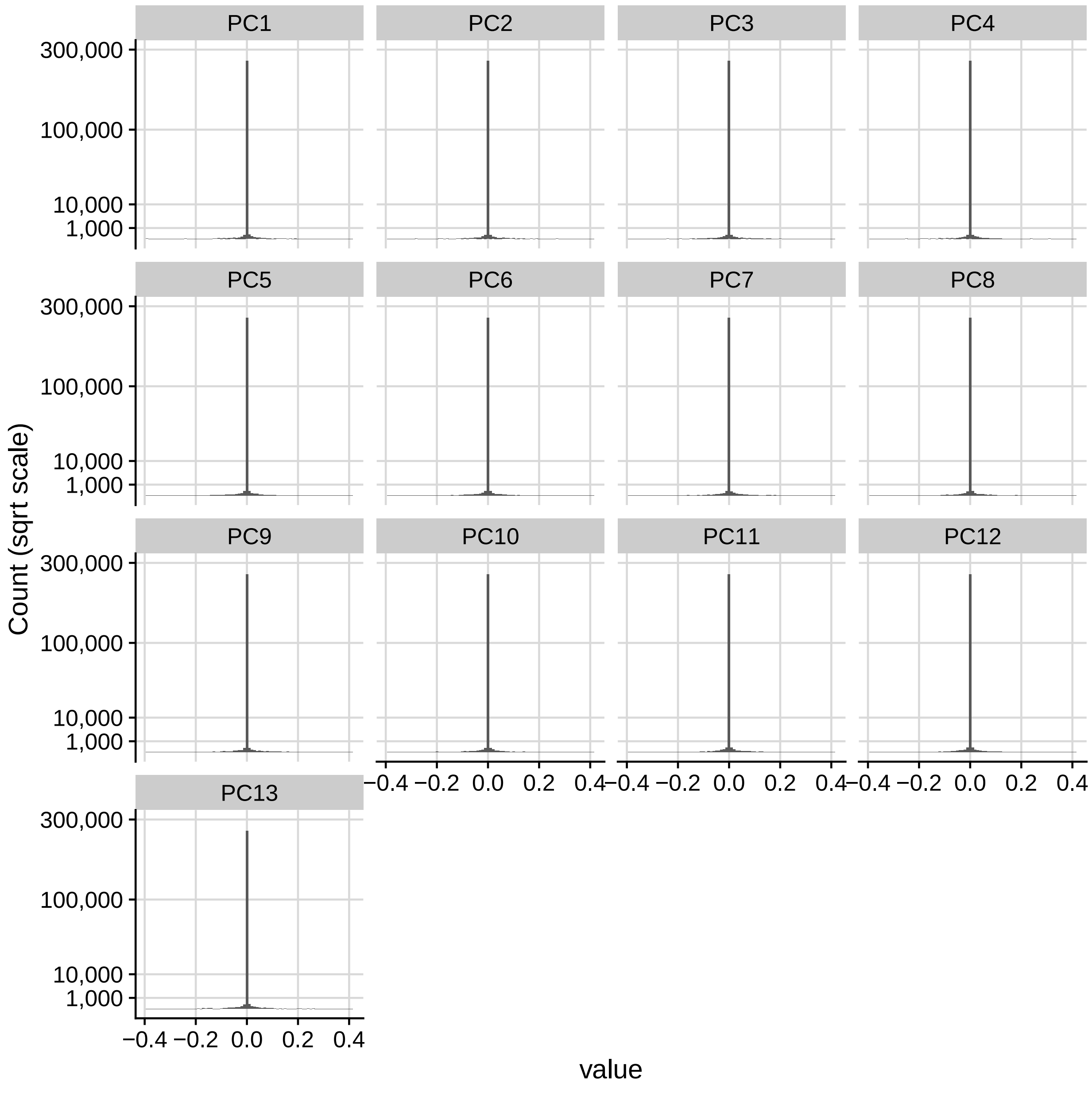

Supplementary Figure 20: Distributions of entries in the rotation matrix for each component PC1-PC13

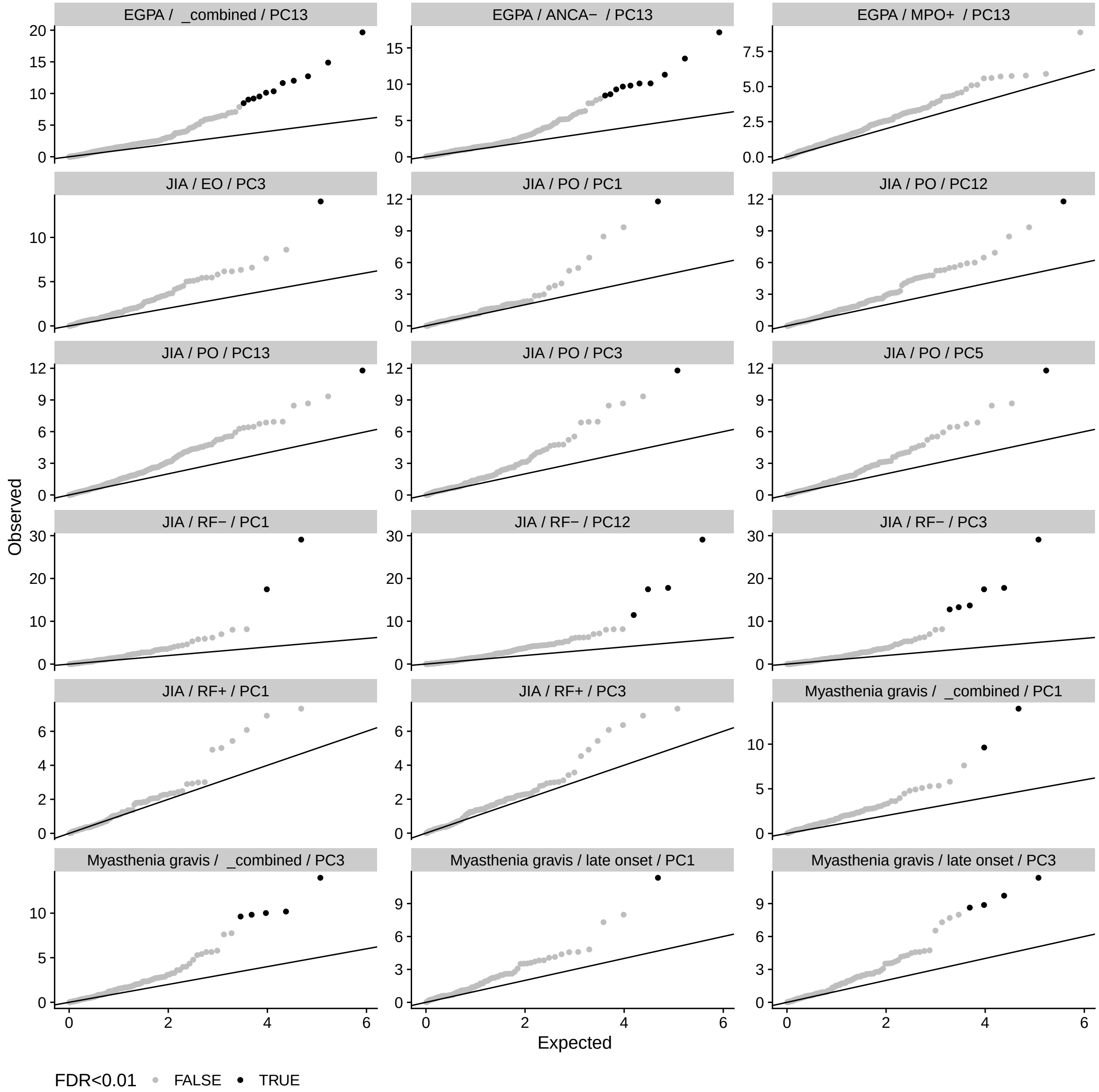

Supplementary Figure 21: QQ plots of p values for driver SNPs on trait-significant components showed a tendency for excess significant results. Points corresponding to FDR $< 1\%$ are highlighted in black, other points in gray. The solid line represents $y = x$.
