# Supplementary material for: Genetic feature engineering enables characterisation of shared risk factors in immune-mediated diseases
Source: Genome Med. 2020 Nov 25;12:106. doi: 10.1186/s13073-020-00797-4 (PMC7687775; doi:10.1186/s13073-020-00797-4)
Supplement: Supplementary file 4 — Additional file 4. Methods for GWAS analysis of individual level datasets: vasculitis, JIA and PsA. [file 13073_2020_797_MOESM4_ESM.docx]

**Vasculitis GWAS analysis**

AAV belongs to a group of IMD characterised by inflammation of the small and medium-sized blood vessels with evidence of circulating pathogenic autoantibodies. It comprises three main syndromes: granulomatosis with polyangiitis (GPA), microscopic polyangiitis (MPA) and EGPA. The two primary antigenic targets of ANCA are proteinase 3 (PR3) and myeloperoxidase (MPO). Although PR3-ANCA is the predominant serotype in GPA and MPO-ANCA is more commonly found in MPA, there is a significant overlap between these syndromes.

The vasculitis cohort we used was part of the discovery cohort from the AAV GWAS performed by the European Vasculitis Genetics Consortium (Lyons et al., 2012), comprising 478 PR3-AAV cases, 264 MPO-AAV and 5,259 controls from the Wellcome Trust Case Control Consortium. All cases had a clinical diagnosis of either GPA or MPA according to the European Medicines Agency algorithm, supported by a positive ANCA assay. The genotyping, calling, and data QC have been previously described (Lyons et al., 2012). Briefly, the genotyping was performed by AROS Applied Biotechnology (Arthus, Denmark) using the Affymetrix SNP6 platform. Pre-phasing and genome-wide SNP imputation were performed using Eagle2 and Minimac3 respectively on the Michigan Imputation Server v1.0.3 that facilitates access to the HRC reference panel (HRC version r1.1 2016) (Das et al., 2016). Post-imputation, SNPs with MAF < 0.01 or r2 < 0.3 were removed from dataset using BCFtools version 1.2, leaving a total of 7,656,576 SNPs available for case-control association testing using a linear mixed model with BOLT-LMM software v2.3.2 (Loh et al., 2015, 2018).

**JIA and PsA GWAS analysis**

The JIA and PsA GWAS datasets were generated and QC’d using the same strategies.

Genotyping and statistical quality control: JIA and PsA DNA samples were genotyped on the Illumina Infinium CoreExome genotyping array in accordance to the manufacturer’s instructions at the Centre for Genetics and Genomics Versus Arthritis (The University of Manchester). Genotype calling was performed by the GenCall algorithm in the GenomeStudio Data Analysis software platform (Genotyping Module v1.8.4). Preliminary genotype clustering was performed using the default Illumina cluster file to identify poor quality samples (call rate < 0.90). Following the exclusion of low-quality samples automated reclustering was performed to calibrate genotype clusters based on the study samples. Sample-level quality control (QC) was performed based on the following exclusion criteria: final call rate < 0.98, outlier based on autosomal heterozygosity (2 standard deviations from the mean) and discrepancy between genetically inferred sex and database records. SNPs were excluded if they were non-autosomal, call rate < 0.98 or a minor allele frequency < 0.01. PsA was compared with 4596 controls from the WTCCC2 study (REF).

JIA was compared with 9,965 population controls from the UK Household Longitudinal Study (https://www.understandingsociety.ac.uk/) accessed via the European Genotype-phenome Archive. Samples were genotyped at the Wellcome Trust Sanger Institute using the Illumina Infinium CoreExome genotyping array. Sample and SNP QC is consistent with that described above for case samples.

Case and control datasets were combined retaining the intersection of SNPs. Identity-by-descent was used to identify related individuals (kinship coefficient > 0.0884) across all study samples performed with the KING software package (version 1.9). For each related pair the sample with the highest call rate was preferentially retained. Individuals were excluded if they were identified as outliers based on ancestry using principal component analysis (PCA) performed with the flashpca software package (version 2.0) where outliers were identified using aberrant R library (version 1.0).

**Imputation**: Prior to imputation SNPs with ambiguous alleles (C/G and A/T) were excluded and remaining SNPs were aligned to the Haplotype Reference Consortium (HRC) panel (version 1.1) using the HRC imputation preparation tool (https://www.well.ox.ac.uk/~wrayner/tools/). Imputation was performed using the Michigan Imputation server where phasing was performed with Shapeit2 and the HRC panel. Following imputation SNPs were excluded based on a MAF < 0.01 and imputation accuracy (r2) < 0.4.

**Association testing, PsA**: case-control association testing was performed using the SNPTEST software package (version 2.5.2) using the score method to account for imputation uncertainty. Three principal components, calculated as described above, were included as covariates to account for any residual population structure.

**Association testing, JIA**: case-control association testing was performed using the snp.rhs.estimates function in the R package SnpStats, comparing in turn overall, or JIA subtypes to the control group. Three principal components, calculated as described above, were included as covariates to account for any residual population structure.

## 
